# PD-1/CTLA-4 Blockade Inhibits Epstein-Barr Virus-Induced Lymphoma Growth in a Cord Blood Humanized-Mouse Model

**DOI:** 10.1371/journal.ppat.1005642

**Published:** 2016-05-17

**Authors:** Shi-Dong Ma, Xuequn Xu, Richard Jones, Henri-Jacques Delecluse, Nicholas A. Zumwalde, Akshat Sharma, Jenny E. Gumperz, Shannon C. Kenney

**Affiliations:** 1 Department of Oncology, School of Medicine and Public Health, University of Wisconsin-Madison, Madison, Wisconsin, United States of America; 2 Department of Medical Microbiology and Immunology, School of Medicine and Public Health, University of Wisconsin-Madison, Madison, Wisconsin, United States of America; 3 Department of Medicine, The University of Texas, MD Anderson Cancer Center, Houston, Texas, United States of America; 4 German Cancer Research Centre (DKFZ) Unit F100, Heidelberg, Germany; Institut National de la Santé et de la Recherche Médicale Unit U1074, Heidelberg, Germany; 5 Departments of Oncology and Medicine, School of Medicine and Public Health, University of Wisconsin-Madison, Madison, Wisconsin, United States of America; Baylor College of Medicine, UNITED STATES

## Abstract

Epstein-Barr virus (EBV) infection causes B cell lymphomas in humanized mouse models and contributes to a variety of different types of human lymphomas. T cells directed against viral antigens play a critical role in controlling EBV infection, and EBV-positive lymphomas are particularly common in immunocompromised hosts. We previously showed that EBV induces B cell lymphomas with high frequency in a cord blood-humanized mouse model in which EBV-infected human cord blood is injected intraperitoneally into NOD/LtSz-*scid/IL2R*γ^null^ (NSG) mice. Since our former studies showed that it is possible for T cells to control the tumors in another NSG mouse model engrafted with both human fetal CD34+ cells and human thymus and liver, here we investigated whether monoclonal antibodies that block the T cell inhibitory receptors, PD-1 and CTLA-4, enhance the ability of cord blood T cells to control the outgrowth of EBV-induced lymphomas in the cord-blood humanized mouse model. We demonstrate that EBV-infected lymphoma cells in this model express both the PD-L1 and PD-L2 inhibitory ligands for the PD-1 receptor, and that T cells express the PD-1 and CTLA-4 receptors. Furthermore, we show that the combination of CTLA-4 and PD-1 blockade strikingly reduces the size of lymphomas induced by a lytic EBV strain (M81) in this model, and that this anti-tumor effect requires T cells. PD-1/CTLA-4 blockade markedly increases EBV-specific T cell responses, and is associated with enhanced tumor infiltration by CD4+ and CD8+ T cells. In addition, PD-1/CTLA-4 blockade decreases the number of both latently, and lytically, EBV-infected B cells. These results indicate that PD-1/CTLA-4 blockade enhances the ability of cord blood T cells to control outgrowth of EBV-induced lymphomas, and suggest that PD-1/CTLA-4 blockade might be useful for treating certain EBV-induced diseases in humans.

## Author Summary

EBV is a human herpesvirus that remains in the host for life, but is normally well controlled by the host immune response. Nevertheless, EBV causes lymphomas in certain individuals, particularly when T cell function is impaired. Antibodies against two different inhibitory receptors on T cells, PD-1 and CTLA-4, have been recently shown to improve T cell cytotoxic function against a subset of non-virally associated tumors. Here we have used an EBV-infected cord blood-humanized mouse model to show that EBV-infected lymphomas express both the PD-L1 and PD-L2 inhibitory ligands for PD-1. Importantly, we find that the combination of PD-1 and CTLA-4 blockade decreases the growth of EBV-induced lymphomas in this model, and demonstrate that this anti-tumor effect requires T cells and enhances their responses to EBV. Our results suggest that PD-1/CTLA-4 blockade might be useful for treating certain EBV-associated diseases in humans.

## Introduction

The human herpesvirus, Epstein-Barr virus (EBV), causes the clinical syndrome, infectious mononucleosis, but is usually well controlled by the host after recovery from the initial infection. However, in a small number of infected patients, EBV contributes to the development of a variety of different B-cell and epithelial-cell malignancies, including Burkitt lymphoma (BL), Hodgkin lymphoma (HL), lymphoproliferative disease (LPD) in immunocompromised hosts, diffuse large B cell lymphomas (DLBCL), undifferentiated nasopharyngeal carcinoma and gastric cancer [1, 2]. In addition, EBV causes poorly controlled, chronic active infection of B cells and/or T cells in rare individuals. A vigorous host immune response is required to control EBV infection, and a number of genetic mutations affecting the functions of various components of the immune system, including T cells, NK cells, and NKT cells, have been shown to contribute to inadequate control of EBV infection [3, 4]. EBV specific T cells are particularly important for preventing and treating EBV-induced lymphoproliferative disease. In fact, donor-derived T cells that recognize donor-derived EBV-transformed B cell lines can be expanded *in vitro*, and used to treat EBV-induced lymphoproliferative disease when injected in bone marrow transplant recipients [5]. Both CD4 and CD8 T cells contribute to this T cell anti-tumor effect, and latent as well as lytic viral antigens are targeted [6].

Certain EBV-positive malignancies, including HL, gastric cancer and nasopharyngeal carcinoma (NPC), commonly occur in seemingly immunocompetent hosts. Since these malignancies express a subset of EBV latency proteins, including EBNA1, LMP1 and LMP2A, it is not clear why the host immune response in such patients cannot recognize and kill the EBV-infected tumor cells. Furthermore, HL and NPC tumors are typically infiltrated by a large number of inflammatory cells that apparently are unable to kill the tumor cells (and may in fact support their growth) [7–12]. Whether the host immune cells surrounding the EBV positive tumor cells might be capable of killing the tumor cells under certain conditions is not currently known.

A number of recent studies have revealed that tumor-infiltrating T cells often express inhibitory receptors that lead to an “exhausted” T cell phenotype that prevents tumor cell killing. Both the CTLA-4 and PD-1 receptors can inhibit T cell receptor (TCR) mediated signaling and T cell activation [13]. CTLA-4 inhibits TCR signaling by competing with the CD28 co-stimulatory receptor for interaction with the CD80 and CD86 ligands expressed on antigen presenting cells [14]. When the PD-1 receptor interacts with either of two different ligands (PD-L1 and PD-L2), it promotes a signal that induces expression of cellular phosphatases (such as SHP1/2) that reverse the tyrosine kinase activity of the TCR signal [15, 16]. Tumor cells often express the PD-L1 and/or PD-L2 ligands, and thus can down-regulate T cell function through activation of the PD-1 receptor [17, 18]. In addition to tumor cells, PD-L1 and/or PD-L2 have also been reported to be expressed on cells infected by certain types of viruses [19]. Of note, EBV-infected NPC cells as well as EBV-infected lymphoma cells have been reported to express PD-L1 [18, 20–23]. Furthermore, lupus patients have been reported to have poor control of EBV infection due to PD-1 mediated cytotoxic T cell exhaustion [24]. Thus, EBV-positive tumors might be relatively resistant to T cell mediated killing due to their ability to activate the PD-1 signaling pathway in nearby T cells.

Monoclonal antibodies which block the CTLA-4 or PD-1 receptors have been recently approved by the FDA for treatment of certain forms of human cancers [13, 25–28]. These blocking antibodies, which are thought to reduce tumor size by increasing the ability of T cells to recognize and kill tumor cells, produce impressive anti-tumor effects in a subset of human tumor types, including melanoma, non-small cell lung cancer, colon cancer, and renal cancer, although only a portion of patients respond to such therapy and biomarkers that predict anti-tumor response have yet to be well defined [29–31]. There is also evidence that the combination of both CTLA-4 antibodies and PD-1 antibodies results in better tumor outcomes than either antibody type alone [32, 33]. The reversal of the exhausted T cell phenotype by CTLA-4/PD-1 blockade is thought to promote T cell mediated killing of tumor cells via T cell recognition of neoantigens that are unique to the tumor cells, and which arise from tumor cell- specific gene mutations. Furthermore, tumors which have a higher number of cellular mutations are more likely to respond to CTLA-4/PD-1 blockade, since there is a higher likelihood that one or more of the tumor mutations will produce a novel epitope which can be recognized by T cells [34, 35].

Given that virally infected malignancies express viral antigens, it seems likely that these tumors might be amongst the most highly susceptible to T-cell mediated killing following CTLA-4-1/PD-1 blockade. NOD/LtSz-*scid/IL2R*γ^null^ (NSG) mice reconstituted with human immune cells provide an excellent small animal model for studying whether manipulation of engrafted human T cell function affects the ability of EBV to induce human B cell lymphomas. In previous studies, we showed that EBV infection in cord blood-humanized NSG mice induces EBV-positive DLBCLs in the majority of mice, although the lymphomas are initially highly infiltrated with T cells [36]. In another humanized NSG mouse model engrafted with both human fetal CD34+ cells and human thymus and liver, we and others showed that it is possible for T cells to control the tumors [37–40]. Here we demonstrate that treatment of EBV-infected cord-blood humanized NSG mice with the combination of both CTLA-4 and PD-1 blocking antibodies strikingly decreases the growth rate of EBV-induced lymphomas, and increases the length of survival in mice. Furthermore, we show that CTLA-4/PD-1 blockade increases the ability of T cells from EBV-infected mice to produce interferon in response to EBV antigens *in vitro*, enhances T cell infiltration into EBV-induced lymphomas, and increases the number of tumor-associated high endothelial venules (a specialized endothelial cell type required for T cell migration into tumors) [41–43]. These results suggest that CTLA-4/PD-1 blockade may be useful for treating certain EBV-associated diseases in humans.

## Results

### Engrafted cord blood T cells have some ability to inhibit growth of EBV-induced lymphomas in the NSG-cord blood model

We previously reported that almost all NSG mice injected i.p. with EBV- infected cord blood develop EBV-positive DLBCLs within 1–2 months after injection [36]. In this model, CD34-depleted cord blood mononuclear cells (including both B cells and T cells) are infected briefly with EBV *in vitro* (1.5 hour) and then injected i.p. into NSG mice. Following i.p. injection, both B cells and T cells are engrafted into the spleen and lymph nodes of mice. EBV-infected (but not mock-infected) cord blood-engrafted mice eventually develop DLBCLs (most commonly involving the pancreas, liver and mesenteric lymph nodes) that become grossly visible 3 to 4 weeks after injection of cells, and then grow very rapidly over a 7–10 day period before mice need to be euthanized. The EBV-infected DLBCLs are initially infiltrated with human T cells, and support the most transforming form of EBV latency (type III), in which 9 viral genes are expressed [36]. Although freshly isolated human umbilical cord blood T cells are naive, we have observed that they become activated to proliferate after transfer into the NSG mice, which is associated with acquisition of effector functions.

**Fig 1:**
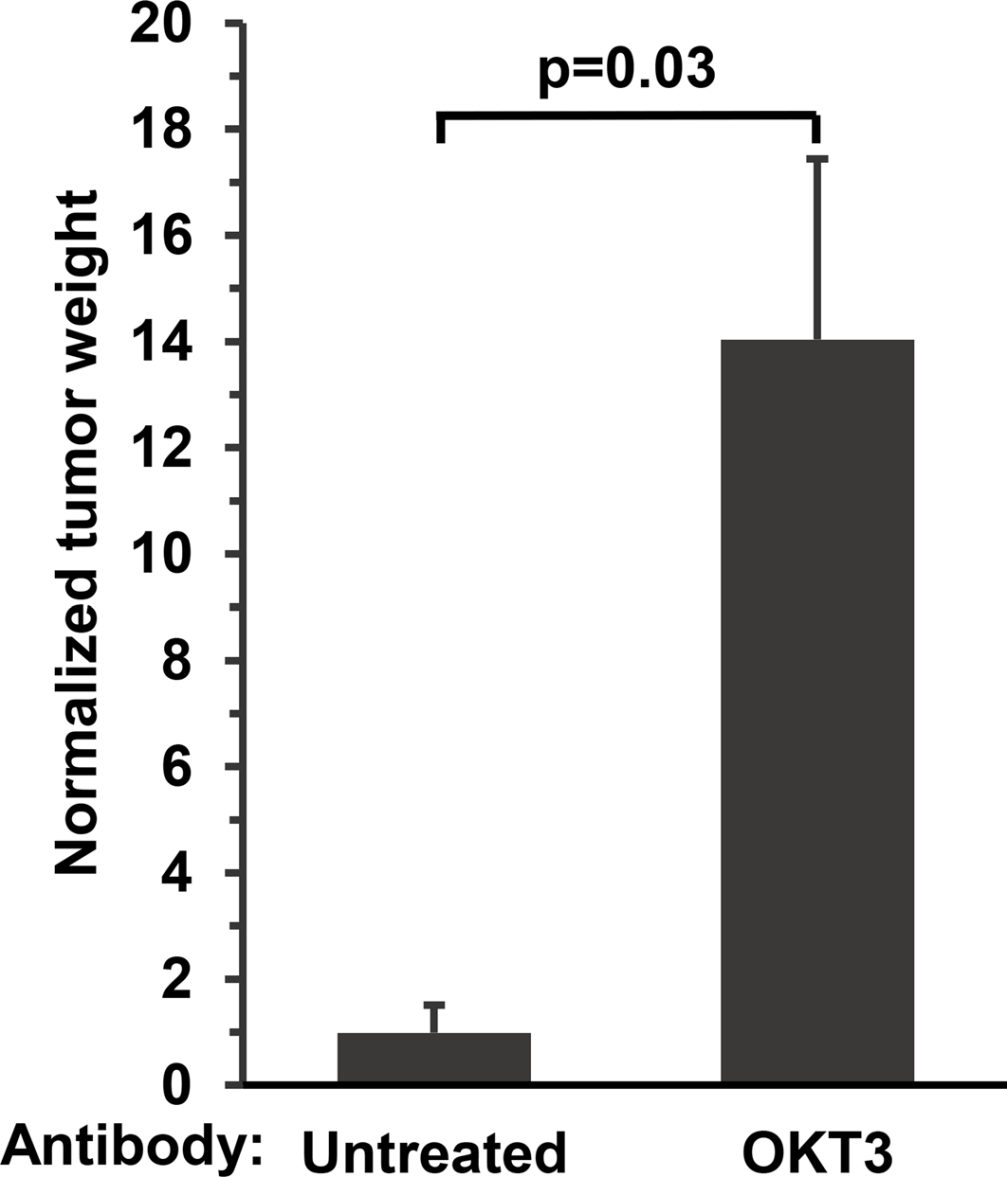
T cells inhibit the growth of EBV-infected B cells in cord blood-humanized mice. NSG mice were injected i.p. with EBV-infected cord blood cells and then treated with or without the OKT3 T-cell depleting ab (50 μg i.p. three times a week starting 11 days after injection of cells, 6 mice/group). Mice were euthanized 26 days post-injection. Grossly visible lymphomas were dissected and weighed. The weight of tumors is normalized to the average size of the untreated tumors (set as 1).

Since both CD8-positive and CD4-positive T cells are engrafted in this model, and both type of T cells infiltrate the EBV-induced DLBCLs, we hypothesized that these T cells might be acting to slow the growth of EBV-induced lymphomas, even if the T cell response to EBV in this model is usually not sufficient to prevent lymphoma growth. To determine if this is the case, NSG mice injected with EBV-infected cord blood were treated with or without a T cell depleting monoclonal antibody (OKT3), starting 5 days after cord blood injection, in order to inhibit engrafted T cell function. As shown in Fig 1, treatment with the OKT3 antibody dramatically increased the size of the EBV-induced lymphomas, suggesting that the presence of the T cells is associated with at least partial control of tumor growth in this model. We therefore hypothesized that the ability of these T cells to control the EBV-driven lymphomas *in vivo* might be limited by the inhibitory (“checkpoint”) ligands in the tumor microenvironment.

### EBV-infected DLBCLs express inhibitory ligands, PD-1, PD-L1 and PD-L2, in cord blood-engrafted NSG mice

**Fig 2:**
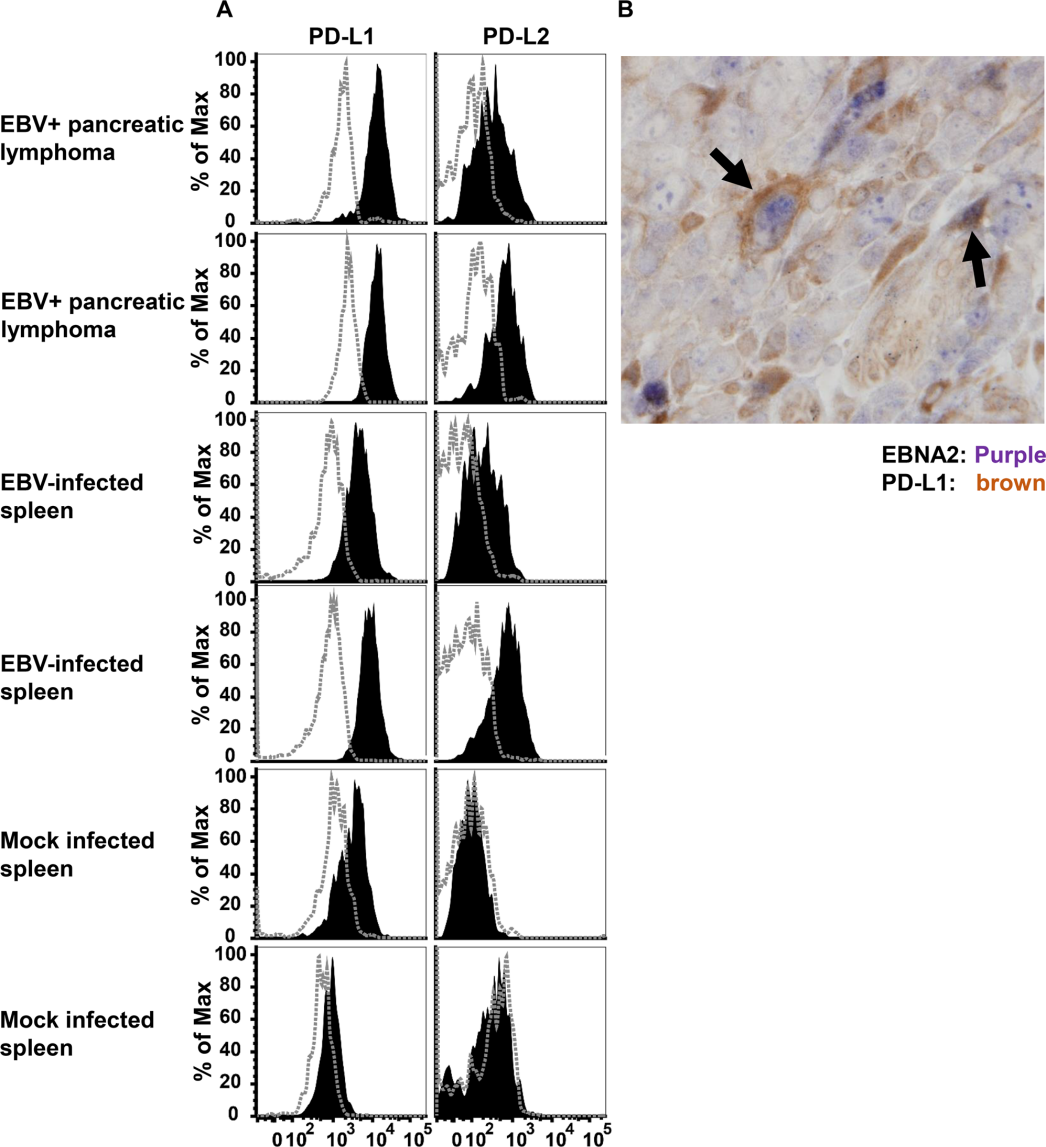
EBV-infected lymphoma cells express the PD-L1 and PD-L2 ligands in cord blood-humanized mice. A) B cells isolated from pancreatic tissue or spleens of mice injected with EBV-infected cord blood, or mock-treated cord blood, were stained with antibodies specific for human CD45, CD19, CD20, CD3, PD-L1, PD-L2 or isotype matched negative controls and analyzed by flow cytometry. Samples were gated on lymphocytic cells expressing human CD45, CD19, and CD20 (B cells). Filled histograms show staining for PD-L1 (left column) or PD-L2 (right column), in comparison to staining of the same population of cells by the isotype control (dashed histograms). B) EBV–infected lymphoma cells in a cord blood-humanized mouse were co-stained for EBNA2 (purple) and PD-L1 (brown) by IHC. Examples of co-staining cells are indicated with black arrows.

We next asked if EBV-infected lymphoma cells express PD-L1 or PD-L2 ligands in cord-blood engrafted NSG mice. Flow cytometry was used to quantitate PD-L1 and PD-L2 expression on the surface of B cells purified from two different pancreatic lymphomas, two different EBV-infected (non-lymphomatous) spleens, or from two different spleens in mice engrafted with mock-infected cord blood cells derived from the same donor. As shown in Fig 2A, both PD-L1 and PD-L2 were expressed on the surface of pancreatic lymphoma cells, and (to a lesser extent) EBV-infected splenic B cells in cord blood-humanized mice infected with the B95.8 strain of EBV. Mock-infected splenic B cells expressed lower levels of PD-L1 and little or no detectable PD-L2 in comparison to the EBV-infected lymphoma cells. Cord blood- humanized animals infected with another strain of EBV, M81, likewise expressed both PD-L1 and PD-L2 on EBV-infected B cells (S1 Fig). We also performed immunohistochemistry to examine PD-L1 and EBNA2 (a latent EBV protein) co-expression on a DLBCL invading the pancreas. As shown in Fig 2B, some EBNA2 expressing lymphoma cells clearly expressed PD-L1 on the surface.

### T cells express the PD-1 and CTLA-4 receptors in EBV-infected cord-blood engrafted NSG mice

**Fig 3:**
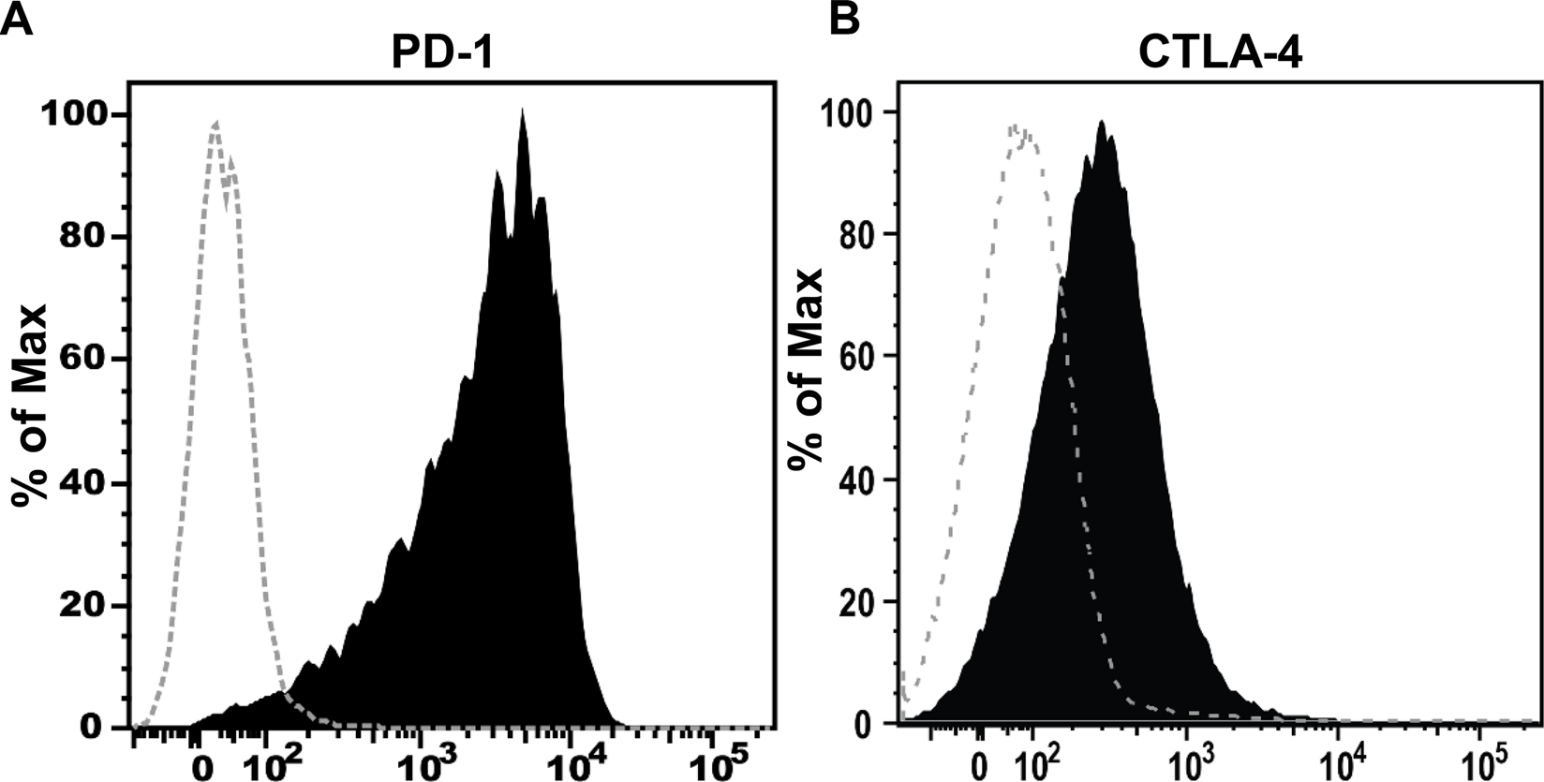
T cells express the PD-1 and CTLA-4 receptors in EBV-infected cord blood-humanized mice. T cells isolated from spleens of EBV-infected cord blood-humanized mice were stained with anti-PD-1 ab **(A),** CTLA-4 **(B)** or isotype control, and analyzed by flow cytometry. Filled histograms show staining for PD-1 **(A),** or CTLA-4 (**B**), in comparison to staining of the same population of cells by the isotype control (dashed histograms). Similar results were obtained in multiple mice and representative results are shown.

We next performed flow cytometry on T cells isolated from the spleens of EBV-infected cord blood humanized mice. As shown in Fig 3A, PD-1 was clearly expressed on the surface of T cells in this model. These results suggest that interactions between the PD-L1 and PD-L2 ligands expressed on EBV-infected lymphoma cells and the PD-1 receptor expressed on T cells might inhibit the ability of T cells to control the growth of EBV-infected lymphoma cells in this model, and that blockade of this interaction with PD-1 blocking antibody might thus improve the ability of cord blood T cells to inhibit lymphoma growth. Likewise, we found that the CTLA-4 receptor was expressed on the surface of T cells in EBV-infected cord blood-humanized mice (Fig 3B), suggesting that blockade of this inhibitory receptor on the tumor-infiltrating T cells might also enhance T cell control of tumor growth.

### The combination of PD-1 and CTLA-4 blocking antibodies inhibits the growth of EBV-induced lymphomas in cord blood-humanized NSG mice

**Fig 4:**
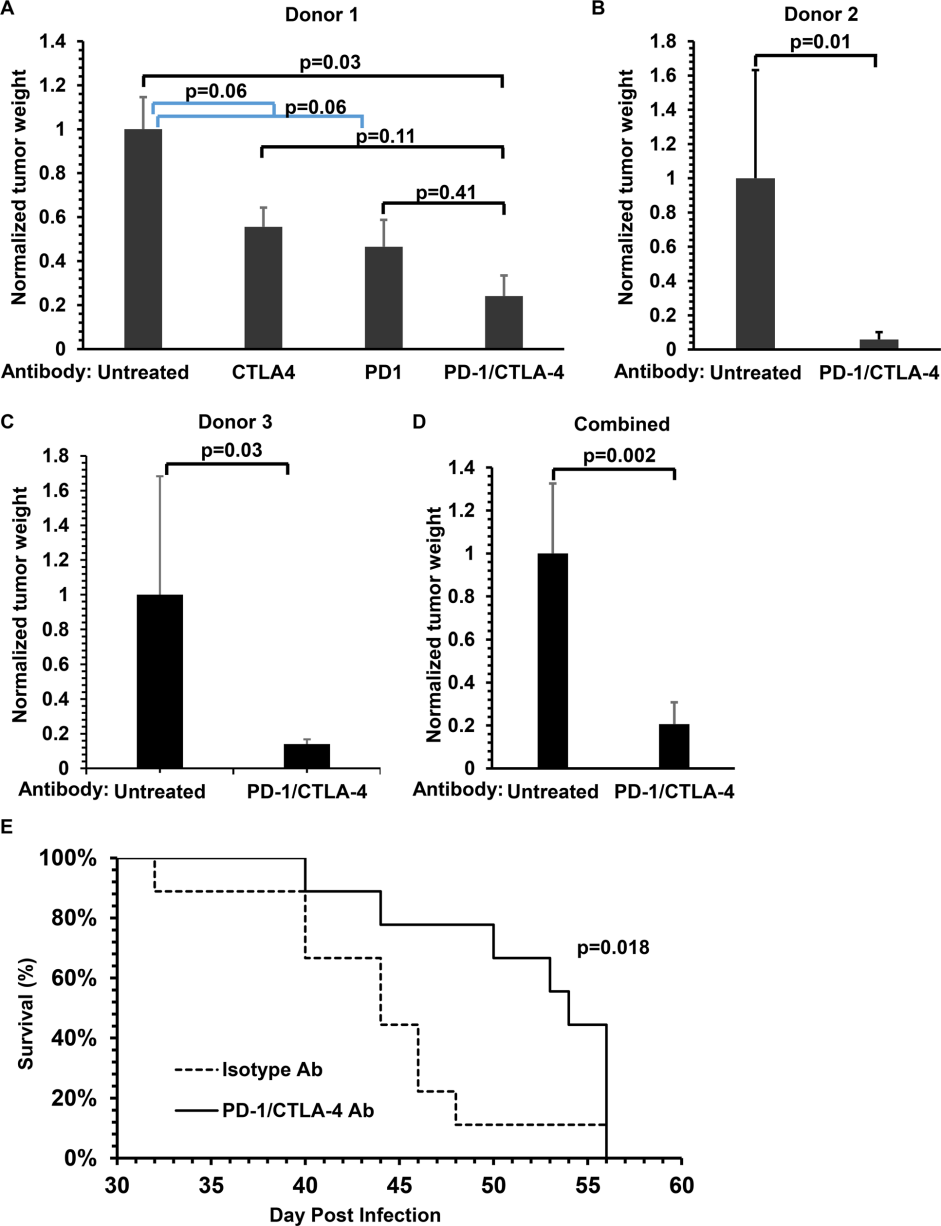
Combined PD-1/CTLA-4 blockade inhibits the growth of EBV-induced lymphomas in cord blood-humanized mice. **A)** M81 strain (2000 infectious units) EBV- infected cord blood-humanized NSG mice were treated with PBS (untreated), anti-PD-1 ab (anti-CD279 J116 mouse monoclonal), anti-CTLA-4 ab (ipilimumab), or anti-PD-1 and anti-CTLA-4 abs as indicated (starting 5 days post infection, 100 μg/animal i.p. 3x/week). Mice were euthanized at 4 weeks after cord blood injection and grossly visible tumors were weighed. The tumor weight is shown for each condition (normalized to the average tumor weight of untreated animals, 4 mice/group). **B and C)** Similar experiments were performed in NSG mice injected with M81 EBV strain-infected cord blood derived from two additional cord blood donors; mice were treated with PBS or the combination of both abs as described above (6 mice/group). **D**. Data from each of the three experiments shown in figure A-C was combined and analyzed. **E**. The survival curves of EBV- infected cord blood-humanized NSG mice treated with or without anti-PD-1/CTLA-4 abs (9 animals each group, infected with 500 infectious M81 EBV units) are shown.

To determine if PD-1 blockade, with or without CTLA-4 blockade, enhances the ability of cord blood T cells to control EBV-induced lymphomas in cord blood-humanized NSG mice, mice injected with EBV-infected cord blood were treated with or without monoclonal antibodies directed against the PD-1 or CTLA-4 receptors, alone or in combination (100 μg per mouse i.p. delivered three times a week starting 5 days after injection of cord blood cells). As shown in Fig 4A, the combination of both PD-1 and CTLA-4 blocking antibodies significantly reduced the size of EBV-induced DLBCLs. Furthermore, the anti-tumor effect of both antibodies together was greater than that of either antibody alone. Therefore, subsequent experiments used the combination of both antibodies.

To determine if PD-1/CTLA-4 blockade inhibits EBV-induced lymphoma growth in NSG mice infected with EBV-infected cord blood derived from more than one donor, NSG mice were injected with EBV-infected cord blood derived from two additional donors, and then treated with or without the CTLA-4/PD-1 antibody combination. As shown in Fig 4B–4D, similar results were obtained in NSG mice injected with EBV-infected cord blood from two additional donors. Therefore, the ability of PD-1/CTLA-4 blockade to enhance cord blood-derived T cell’s ability to control EBV-induced DLBCLs is donor independent. Similar results were also obtained when the initiation of PD-1/CTLA-4 blockade was delayed until 10 days after the injection of EBV-infected cord blood, and the “control” animals were treated with appropriate isotype control antibodies (S2 Fig). In addition, a survival study confirmed that the length of survival in EBV-infected cord blood humanized mice was significantly longer in PD-1/CTLA-4 treated animals, although all animals eventually succumbed to lymphoma (Fig 4E).

### PD-1/CTLA-4 blockade enhances the ability of T cells to respond to EBV antigens *in vitro*, and T cells are required for its anti-tumor effect *in vivo*

**Fig 5:**
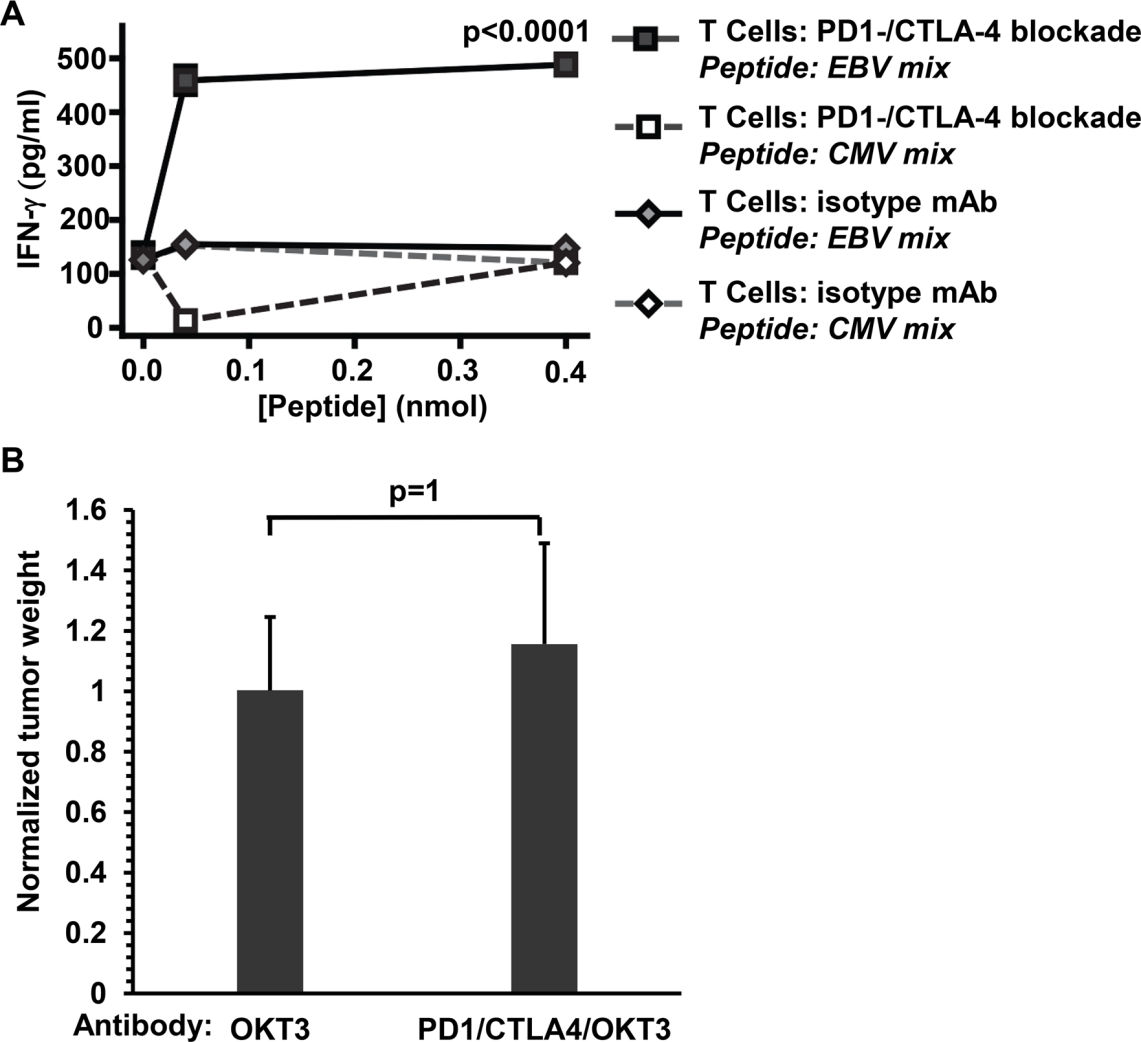
T cells are required for the therapeutic effect of PD-1/CTLA-4 blockade in EBV-infected humanized mice. **A.** Human T cells were harvested at 4 weeks post-injection from spleens of EBV- infected mice that were PD-1/CTLA-4 Ab treated, or treated with istoype control Abs. The T cells were incubated for 72 hr in medium containing IL-2, then exposed to autologous umbilical cord mononuclear cells in the presence of vehicle control (shown as a peptide concentration of "0"), a mixture of synthetic EBV peptides (“EBV peptide”), or a mixture of CMV peptides (“CMV peptide”), and IFN-γ secreted into the culture supernatant was quantified by ELISA. The plot shows the means of 3–6 replicates for each condition with error bars indicating the standard deviations. The p value shown (calculated by a 2-tailed student's t test) is for the IFN-γ values produced by T cells against the EBV peptide. **B**. NSG mice were injected i.p. with EBV-infected cord blood cells and then treated with or without anti-PD-1/anti-CTLA-4 antibodies, in the presence of the OKT3 T-cell depleting ab (6 mice/group). Mice were euthanized 26 days post-injection. Grossly visible lymphomas were dissected and weighed. The weight of tumors is normalized to the average size of the tumors not treated with PD-1/CTLA-4-abs (set as 1).

Since activated B cells, in addition to activated T cells, can express PD-1, the therapeutic effect of PD-1/CTLA4 blockade against EBV-induced lymphomas in humanized mice could potentially involve “off-target” effects directed against EBV+ lymphoma cells rather than enhancement of the T cell anti-tumor response. To confirm that PD-1/CTLA-4 antibody treated EBV-infected animals in this humanized mouse model develop EBV-specific T cells, we tested for T cell responses to EBV peptides in EBV-infected animals that were treated with immune checkpoint blockade or isotype control Ab. T cells isolated from spleens of animals that were given the immune checkpoint blockade showed clear IFN-γ responses when exposed to autologous cord blood cells in the presence of a mixture of synthetic EBV peptides, but did not respond to control CMV peptides (Fig 5A). T cells from infected animals that were treated with isotype control Ab showed either weak or no detectable response to synthetic EBV peptides (Fig 5A). T cells from uninfected mice showed no specific response to EBV or CMV peptides (S3 Fig). These results indicate that in the presence of PD-1/CTLA-4 blockade, cord blood derived T cells can give rise to an EBV antigen specific population that produces the anti-tumor cytokine IFN-γ.

To determine if PD-1/CTLA-4 inhibition of EBV-induced tumor growth in cord blood-humanized mice requires the presence of T cells, EBV-infected animals were treated with or without PD-1/CTLA-4 blockade in the presence of the OKT3 T cell depleting antibody. As shown in Fig 5B, in the presence of concomitant OKT3 treatment, PD-1/CTLA-4 ab treatment had no effect on the size of EBV-induced tumors. This result confirms that T cells are required for the ability of PD-1/CTLA-4 antibodies to inhibit EBV-induced lymphomas in cord blood-humanized mice.

### PD-1/CTLA-4 blockade increases T cell infiltration of tumors, and promotes T cell activation

**Fig 6:**
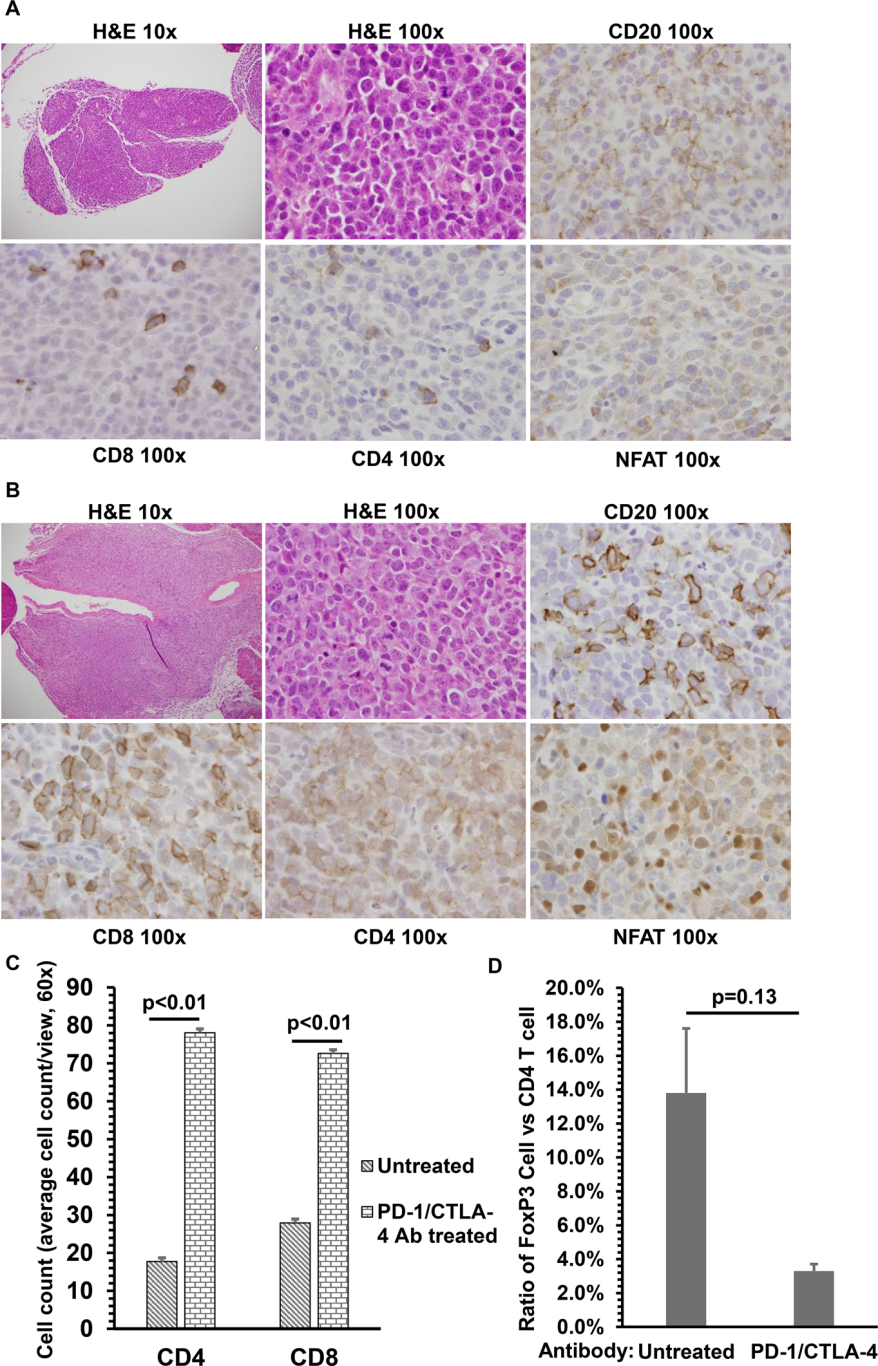
PD-1/CTLA-4 blockade increases T cell tumor infiltration and T cell activation in EBV-infected cord-blood humanized mice. A) Lymphomas from euthanized untreated EBV-infected cord blood-humanized NSG mice were formalin-fixed and paraffin embedded. Tumors were stained with H&E (10x magnification and 100x magnification), or antibodies for CD20 (B cells), CD8 (cytotoxic T cells), CD4 (helper T cells) and NFATC1 (activated T cell) as indicated. No nuclear NFATC1 staining (indicative of T cell activation) was observed. At least 8 mice were analyzed from each condition and representative figures are shown. B) Lymphomas from EBV-infected cord blood-humanized NSG mice treated with PD-1 and CTLA-4 antibodies were formalin-fixed and paraffin embedded. Tumors were stained with H&E (10x magnification and 100x magnification), or antibodies for CD20 (B cells), CD8 (cytotoxic T cells), CD4 (helper T cells) and NFATC1as indicated. At least 8 mice were analyzed from each condition and representative figures are shown. C) The average number of infiltrating CD4 and CD8 T cells in PD-1/CTLA-4 treated versus untreated tumors was quantitated from 5 different views (60X), using three different tumors for each condition. D) The number of infiltrating FoxP3 positive cells, divided by the number of infiltrating CD4 T cells, was quantitated from 5 different views (60X), using three different tumors for each condition.

The reduced size of EBV-induced lymphomas in PD-1/CTLA-4 antibody treated cord-blood humanized mice suggests that cord blood-derived human T cells may be better able to infiltrate lymphomas and kill tumor cells following PD-1/CTLA-4 blockade. To examine the effect of PD-1/CTLA-4 blockade on T cell infiltration of tumors, EBV-induced lymphomas in PD-1/CTLA-4 antibody-treated animals, versus untreated animals, were formalin fixed, paraffin-embedded and then examined by H & E and immunohistochemistry (IHC) staining using antibodies directed against B cell and T cell markers. Only small lymphomas of similar size were examined in each set of animals, since large tumors had little if any T cell infiltration. As shown in Fig 6A, in the absence of PD-1/CTLA-4 antibody treatment, EBV- infected DLBCLs were primarily composed of CD20 B cells, although infiltrating CD8-positive T cells and CD4-positive T cells were present in the smaller tumors. As a marker for T cell receptor (TCR) activation, we also stained tumors with an antibody directed against the NFATC1 protein, since TCR activation results in NFAT transcription factors being more highly expressed and migrating to the nucleus [44]. The EBV-induced lymphomas in untreated animals had little if any nuclear NFATC1 (Fig 6A).

In contrast, lymphomas of similar size in EBV-infected animals treated with PD-1/CTLA-4 blockade had many more infiltrating CD8-positive and well as CD4-positive T cells (Fig 6B), such that T cells often outnumbered the B cells in tumor infiltrates. Furthermore, increased NFATC1 expression, as well translocation of NFATC1 to the nucleus, was observed in the smaller lymphomas in PD-1/CTLA-4 treated animals (Fig 6B). The enhanced CD4/CD8 T cell infiltration into PD-1/CTLA-4 ab treated tumors is quantitated in Fig 6C. We did not observe a significant change in the proportion of CD4 cells expressing Fox P3 (a marker for T_reg_ cells)(Fig 6D), although the treated tumors tended to have a lower proportion of Fox P3-positive cells. Of note, although larger lymphomas were much less common in the PD-1/CTLA-4 treated versus untreated animals, when they did occur in the treated animals they had much reduced T cell infiltration and NFATC1 nuclear accumulation in comparison to the smaller tumors. Thus infiltration of T cells into lymphomas and NFATC1 nuclear translocation strongly correlates with reduced lymphoma size in this EBV-infected cord blood model.

### PD-1/CTLA-4 blockade increases the number of high endothelial venules (HEVs) in lymphomas

**Fig 7:**
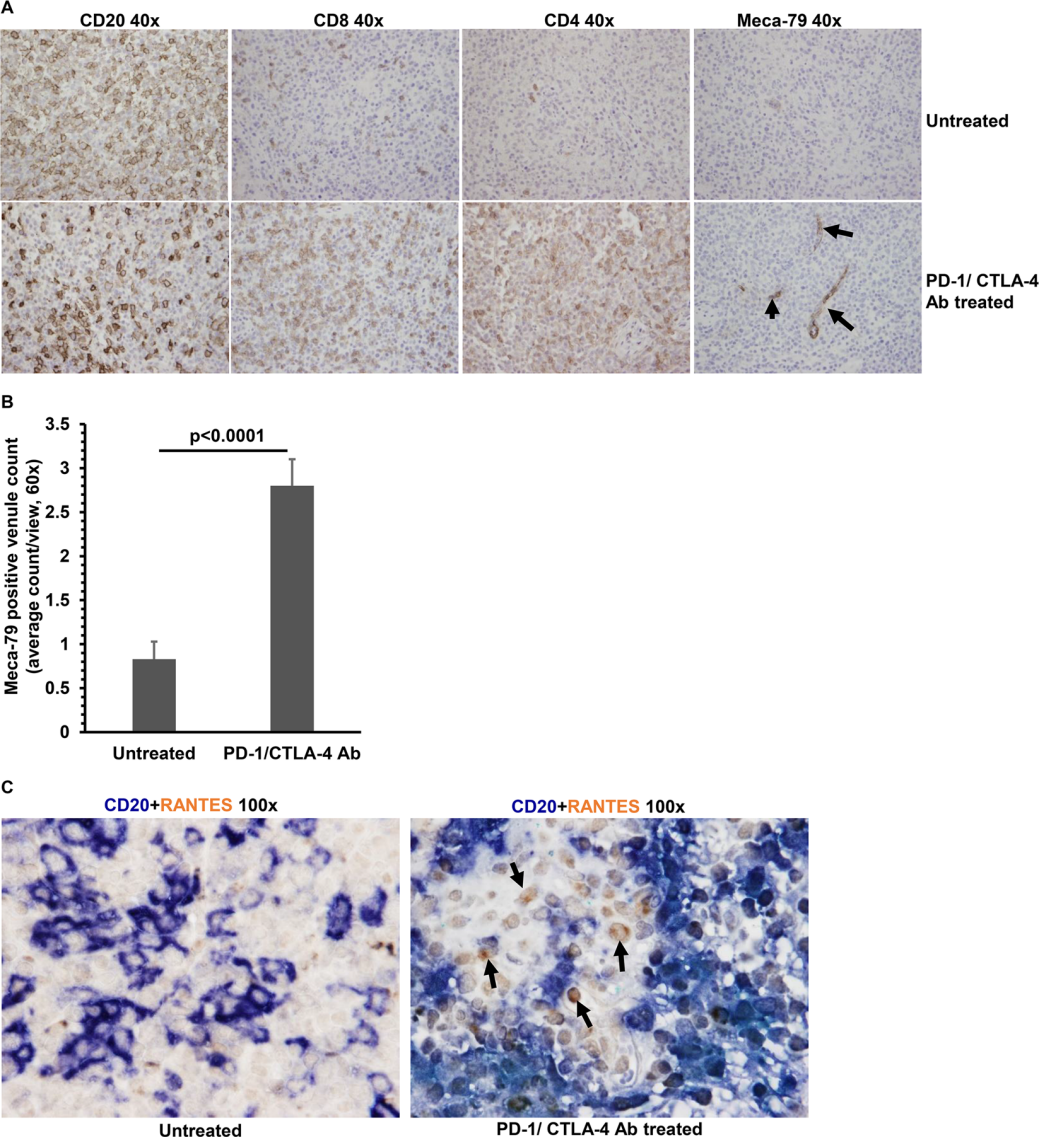
PD-1/CTLA-4 blockade increases the number of intra-tumor high endothelial venules, and enhances RANTES production in tumors. **A).** Lymphomas from EBV-infected cord blood-humanized NSG mice treated with or without PD-1 and CTLA-4 antibodies were formalin-fixed and paraffin embedded, and stained with antibodies for CD20 (B cells), CD8 (cytotoxic T cells), CD4 (helper T cells) and MECA-79 (a marker of high endothelial venules). Arrows are examples of positive staining HEVs. At least 8 mice were analyzed from each condition and representative figures are shown. **B).** The average number of Meca-79 positive venules in PD-1/CTLA-4 treated versus untreated tumors was quantitated from 5 different views (60X), using seven different tumors for each condition. **C).** Lymphomas from EBV-infected cord blood-humanized NSG mice treated with or without PD-1 and CTLA-4 antibodies were formalin-fixed and paraffin embedded, and co-stained with antibodies for CD20 (B cells) and RANTES. Arrows are examples of cells staining positive for RANTES and negative for CD20. At least 8 mice were analyzed from each condition and representative figures are shown.

To further examine potential mechanisms by which PD-1/CTLA-4 blockade promotes T cell infiltration of lymphomas in this model, we determined if immune checkpoint blockade increased the number of high endothelial venules (HEVs) in tumors. HEVs are required for extravasation of T cells into peripheral tissues, including tumors [41–43], and the efficacy of immune checkpoint blockade has been postulated to require the presence of HEVs in tumors [42]. We found that PD-1/CTLA-4 ab-treated lymphomas that contained many infiltrating T cells also had multiple HEVs (Fig 7A and 7B), whereas lymphomas that had few or no infiltrating T cells did not contain these structures (Fig 7A and 7B). Thus, an increased number of intra-tumor HEVs in the PD-1/CTLA-4 treated animals likely plays a role in promoting enhanced T cell infiltration of lymphomas.

In addition, we determined if enhanced T cell infiltration in lymphomas of PD-1/CTLA4-treated animals was associated with increased expression of the T cell chemokine, RANTES, within lymphomas. As shown in Fig 7C, lymphomas in PD-1/CTLA-4 treated animals expressed more RANTES than did the lymphomas in untreated animals. Interestingly, although the EBV LMP1 protein induces RANTES expression [45], the source of RANTES appeared to be primarily from the inflammatory infiltrate rather than the EBV-infected B cells. Thus, increased intra-tumor expression of a potent T cell chemokine may also contribute to increased tumor T cell infiltration in response to immune-checkpoint blockade.

### PD-1/CTLA-4 blockade reduces the number of both latently, and lytically, EBV-infected lymphoma cells in EBV-infected cord blood-humanized mice

**Fig 8:**
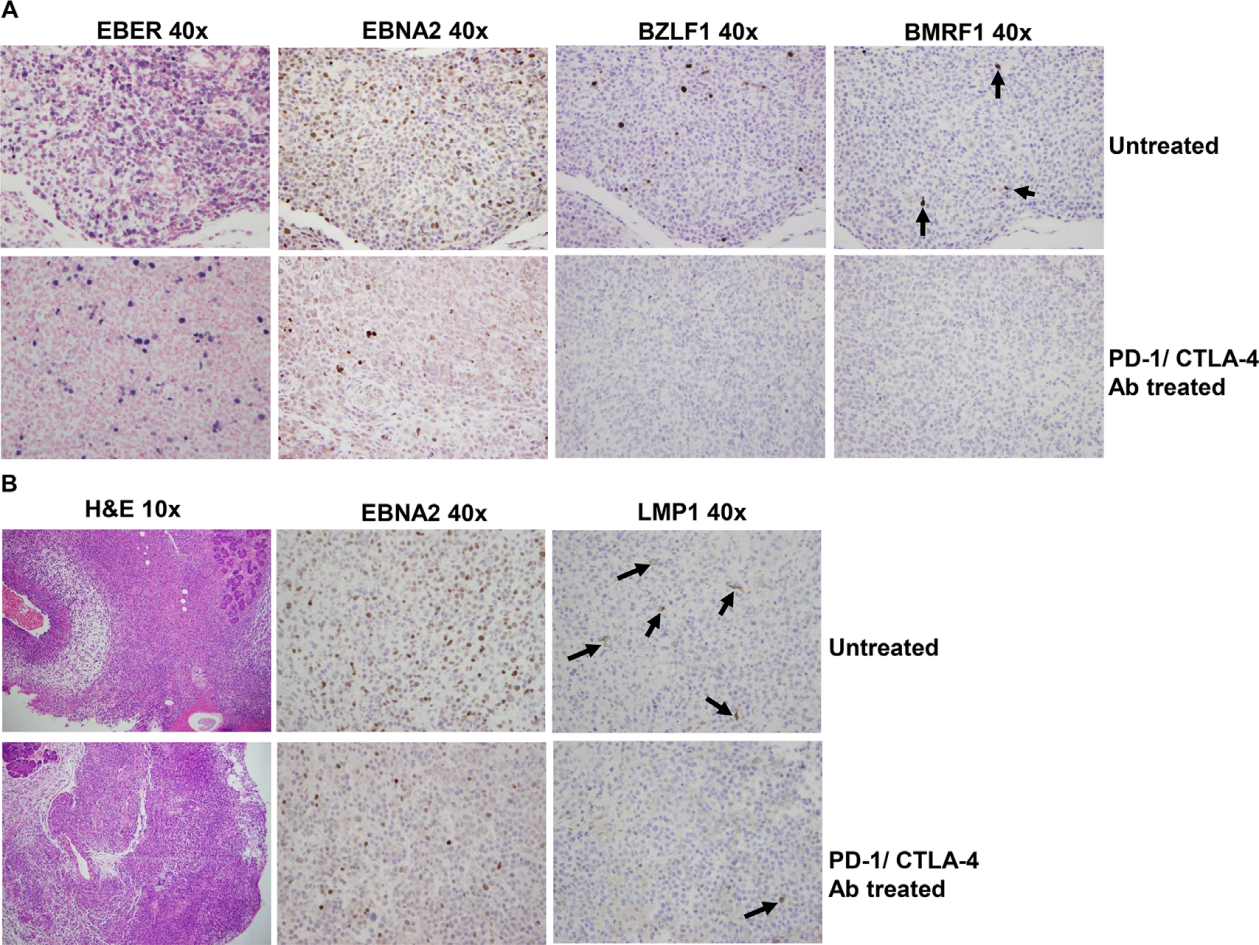
PD-1/CTLA-4 blockade decreases the number of latently, and lytically, EBV-infected B cells in cord-blood humanized mice. A. Lymphomas from EBV-infected cord blood-humanized NSG mice treated with or without PD-1 and CTLA-4 antibodies were formalin-fixed and paraffin embedded. Tumors were examined for EBER expression using ISH, and for expression of EBV latent protein EBNA2, and lytic proteins BZLF1 and BMRF1, using IHC as indicated. Arrows are examples of positively BMRF1-staining cells. At least 8 mice were analyzed from each condition and representative figures are shown. B. Lymphomas from EBV-infected cord blood-humanized NSG mice treated with or without PD-1 and CTLA-4 antibodies were formalin-fixed and paraffin embedded. Tumors were examined for expression of EBV latent proteins EBNA2 and LMP1 using IHC as indicated. Arrows are examples of positively LMP1-staining cells. At least 8 mice were analyzed from each condition and representative figures are shown.

EBV-infected cells that have the lytic form of viral infection express many more viral antigens than latently infected cells, and are thought to be more susceptible to T-cell mediated killing. To determine if PD-1/CTLA-4 blockade preferentially affects the ability of T cells to kill latently- versus lytically- infected lymphoma cells, we examined the number of EBER positive cells by *in situ* hybridization, and performed IHC using antibodies to detect the EBV-encoded EBNA2, LMP1, BZLF1 and BMRF1 proteins. The EBERs are small virally encoded RNAs expressed at high level in all latently EBV-infected cells, the EBNA2 protein is specifically expressed during type III latency, the LMP1 protein is expressed in type II and type III latency, and the BZLF1 and BMRF1 proteins are specifically expressed in lytically infected cells. As shown in Fig 8, both latently and lytically infected lymphoma cells were decreased in EBV-infected cord blood-engrafted animals treated with the PD-1/CTLA-4 blocking antibodies. Thus, PD-1/CTLA-4 blockade may broadly enhance the ability of T cells to recognize a variety of different EBV antigens.

## Discussion

EBV infects the great majority of humans, but is normally well controlled by the host immune response. Nevertheless, even in healthy EBV-infected individuals, the virus persists for life within latently infected B cells, and periodically reactivates to produce infectious viral particles in the saliva. Thus, EBV-infected individuals continue to be at risk for developing EBV-associated diseases and malignancies for their entire lifetime, and a continuous robust T cell response against EBV is required for protection from various different EBV-induced illnesses. An increasingly recognized mechanism by which virally infected cells, as well as tumor cells, can overwhelm the T cell response is to induce T cell “exhaustion” by expressing ligands for the inhibitory T cell receptors, CTLA-4 and PD-1. In this report, we have used a new cord blood-humanized mouse model to show that EBV-infected B cells express inhibitory ligands (PD-L1 and PD-L2) for the PD-1 T cell receptor, and to demonstrate that blockade of the PD-1/CTLA-4 inhibitory T cell receptors enhances the ability of T cells to control growth of EBV-induced lymphomas in this model. These results suggest that PD-1/CTLA-4 blocking antibodies may also be useful for certain types of EBV-induced illnesses in humans.

A variety of mutations that affect various components of the immune response have been reported to decrease control of EBV infection, resulting in persistent high level EBV viremia and in some cases EBV-induced LPD [4]. These syndromes can results from mutations in a variety of different genes, including SH2D1A, XIAP, PI3KCD, PRF1, MAGT1, ITK1, CORO1A and PRKCD [4]. Reduced T cell function also increases the risk of certain types of EBV-induced tumors. For example, AIDS patients are at much higher risk than the general population for developing EBV-infected primary CNS lymphomas, EBV-positive DLBCLs, EBV-positive Hodgkin Disease and EBV-positive Burkitt lymphomas [46–49]. Likewise, organ transplant recipients (particularly EBV-negative individuals), who have reduced T cell function due to iatrogenic immunosuppression, have a high risk for EBV-induced LPD [50, 51]. Children with malaria, who are known to have reduced T cell function [52], often have extremely high levels of EBV DNA in the blood, which may increase their likelihood of eventually developing EBV-positive Burkitt lymphomas [53]. Even relatively subtle immunosuppression, such as occurs during normal aging, may contribute to certain EBV-associated malignancies, in particular age-related EBV-positive diffuse large B cell lymphoma [54]. The EBV-positive tumors that occur in immunocompetent individuals (including gastric carcinoma, NPC, BL and HL) generally express many fewer virally-encoded latency proteins than the latency type III tumors that occur in immunosuppressed patients, and thus are less immunogenic.

Nevertheless, since at least three different EBV proteins (EBNA1, LMP1 and LMP2A) are expressed in both HL and NPC [55, 56], and LMP1 expression in particular has been shown to promote T cell-mediated killing [57–60], a number of studies have investigated the potential mechanism(s) by which T cell-mediated killing of EBV-infected tumor cells might be inhibited in these tumors. In this report, we confirm that EBV infection of B cells increases PD-L1 expression, and show that PD-L2 expression is also increased. Interestingly, although previous reports have identified LMP1 as the likely EBV protein that increases PD-L1 expression, we found that both wildtype M81 strain EBV, and an LMP-1 deleted B95.8 strain EBV mutant [36], activate both PD-L1 and PD-L2 expression on EBV-infected B cells in humanized mice (Fig 2A and S1 Fig). Thus, LMP1 expression is not required for induction of PD-L1/PD-L2 on EBV-infected tumors. Whether tumors with type I latency such as BL (expressing only the viral EBNA1 protein, small EBV nuclear RNAs and virally-encoded miRNAs) also express PD-L1 and/or PD-L2 is currently unknown. However, since both PD-L1 and PD-L2 were recently reported to be expressed in EBV-positive (but not EBV-negative) gastric cancers [61], which often have type I latency, it is possible that even type I latency EBV infection promotes PD-L1 and/or PD-L2 expression on host cells.

To our knowledge, this is the first study to show that PD-1/CTLA-4 blockade decreases the growth of EBV-induced lymphomas in a humanized mouse model. Our finding that PD-1/CTLA-4 blockade in this model greatly enhances the ability of T cells to produce the anti-tumor cytokine IFN-γ in response to EBV peptides confirms and extends a recent *in vitro* study showing that PD-1 blockade increases the ability of co-cultured T cells to proliferate in the presence of EBV-positive DLBCLs [23]. Furthermore, we confirm that the effect of immune checkpoint blockade in the humanized mouse model requires the presence of functional T cells, since this treatment had no effect when T cells were depleted using the OKT3 antibody. Our results suggest that PD-1/CTLA-4 blockade increases the ability of T cells to infiltrate EBV-induced lymphomas, and to become highly activated (as measured by NFATC1 nuclear translocation). Consistent with our results, a previous study in melanoma patients reported that enhanced T cell infiltration of tumors following immune checkpoint blockade was associated with a higher likelihood of clinical response [62].

In addition, we found that enhanced T cell infiltration of PD-1/CTLA-4 ab treated lymphomas was accompanied by a higher number of intra-tumor high endothelial venules (HEVs). HEVs, which are composed of specialized endothelial cells that express the peripheral lymph node “addressin”, are required for T cell entry into tumors (and lymph nodes) and have been proposed to play an essential role for the success of various types of immunotherapy [42]. Given that EBV-infected B cells with type III latency constitutively express a variety of T cell-attracting chemokines, including RANTES [45] and IP10 [63], the ability of tumor endothelial cells to differentiate into HEV structures may act as a critical gate-keeper in controlling tumor T cell infiltration into EBV-induced lymphomas. Since the development of lymph-node like vasculature within tumors is induced by activated T cells and NK cells via secretion of lymphotoxin alpha 3 and interferon gamma [43], PD-1/CTLA-4 blockade may increase the number of HEV structures within tumors by enhancing T cell activation, provided that some T cells are already infiltrating the tumor prior to treatment. Although HEVs likely enhance the ability of EBV-infected B cells to migrate to lymph nodes and lymph-node like structures early in tumor development, since we found that large tumors (which had few if any T cells) no longer contained these specialized endothelial structures, HEVs are not required for continued EBV+ lymphoma growth once tumors become established.

We also found that PD-1/CTLA-4 blockade reduced the number of latently infected, as well as lytically infected, EBV-positive lymphoma cells in this humanized mouse model. Nevertheless, PD-1/CTLA-4 blockade did not prevent the establishment of latent EBV infection, at least when given 5 to 10 days after EBV infection was initiated. Since EBV latency establishment may require expression of only a single viral protein, EBNA1 (which mediates latent viral replication via the host DNA polymerase), and EBNA1 is known to be poorly recognized by the host immune response [64, 65], it is possible that PD-1/CTLA-4 blockade may be less effective at controlling tumors such as BL and gastric carcinoma that have type I latency. Nevertheless, the recent observation that PD-L1 and PD-L2 are expressed in EBV-positive, but not EBV-negative, gastric carcinomas suggests that PD-1/CTLA-4 blockade may be therapeutically useful even for EBV-positive cancers with the less immunogenic forms of viral latency.

Although PD-1/CTLA-4 blockade greatly decreased the growth rate of EBV-induced lymphomas in the cord blood humanized mouse model, and extended the survival of EBV-infected mice, this therapy alone did not ultimately prevent death due to EBV+ lymphoma. The failure to do so may reflect the relative paucity of some aspects of the intact host immune response in this CD34-depleted cord blood model (e.g., NK cells), the absence of EBV-specific memory T cells at the initiation of EBV infection, and the fact that EBV is a highly transforming virus that has many mechanisms for dampening the host immune response. Thus, PD-1/CTLA-4 blockade may be even more effective for controlling EBV-positive lymphomas in the context of certain types of human immunodeficiency (for example, following solid organ transplantation), where EBV-specific memory T cells and/or NK cells are present.

Finally, it is important to note that PD-1/CTLA-4 blockade produces toxic (sometime fatal) side effects in some patients, including autoimmune mediated pulmonary infiltrates [66]. In particular, PD-1/CTLA-4 blockade might cause organ transplant rejection or increase graft versus host disease in organ or bone marrow transplant recipients with EBV-associated lymphoproliferative disease. In addition, since many of the clinical symptoms in infectious mononucleosis are due to an overly exuberant T cell response to EBV-infected B cells, PD-1/CTLA-4 blockade could potentially be more harmful than helpful in such patients. Nevertheless, our results here suggest that PD-1/CTLA-4 blockade may be highly useful for treating EBV-positive tumors in otherwise immunocompetent individuals. Patients with age-related EBV-positive DLBCLs might also benefit from PD-1/CTLA-4 blockade. In addition, PD-1/CTLA-4 blockade might help to control EBV infection in patients with specific defects in host immunity that promote uncontrolled EBV infection, as long as these defects do not totally inhibit T cell function. Although highly promising, treatment of EBV-associated illnesses with PD-1/CTLA-4 blockade in humans will need to be carefully studied in a variety of different types of EBV-induced diseases before it can be generally recommended.

## Materials and Methods

### EBV viruses

Most experiments in this study were performed using the lytic EBV M81 strain bacmid DNA. This M81 bacmid expresses the green fluorescent protein (GFP) and a hygromycin B resistance gene, and was constructed using bacterial artificial chromosome technology as described previously [67]. All studies used the M81 strain EBV except for the PD-L1 and PD-L2 flow cytometry studies shown in Fig 2, which were performed in animals infected with a LMP1-knockout (LMP1-KO) mutant virus derived by mutagenesis of the p2089 B95.8 strain EBV bacmid as previously described [68].

### Humanized NOD/LtSz-*scid/IL2R*γ^null^ mice

Immunodeficient nonobese diabetic/severe combined immunodeficient (NOD/LtSz-*scid/IL2R*γ^null^) mice were purchased from Jackson Labs (catalogue 005557). Commercially purchased CD34-depleted human cord blood mononuclear cells (AllCells, LLC., CB117) were mock-infected, or infected with M81 strain EBV, *in vitro* for 1.5 hours, and then 12 to 25 million cells were injected intraperitoneally (i.p.) into 3–5 week old NSG mice. Each individual treatment experiment (with or without blocking antibodies) was performed in animals injected with the same number of cord blood cells (all from the same donor), and the same amount of infectious M81 virus.

### Production of infectious virus

Infectious viral particles were produced from 293 cell lines stably infected with the M81 strain EBV virus following transfection with EBV BZLF1 expression vector as previously described [38]. EBV was titered on Raji cells using the Green Raji cell assay as previously described [38]. The number of fluorescent Raji cells derived from serial dilutions of the concentrated virus stock was examined to calculate the green Raji unit (GRU) titer. Note that the GRU titer from M81 virus infected cells may be less than the actual infectious titer since the GFP gene (inserted into the terminal repeat region of the EBV genome) is sometimes lost during viral replication due to terminal repeat recombination.

### EBV infection of mice

Cord blood was mock-infected, or infected with 2,000 to 5,000 infectious units of M81 EBV virus for 1.5 hours *in vitro*, and then injected i.p. into NSG mice (diluted in PBS with 200 ul final volume). Cord blood was infected with EBV in batch for each individual experiment, divided into aliquots, and antibody treated, versus untreated, animals in each experiment were injected with the same number of EBV-infected cord blood cells, all derived from the same donor. Mice were sacrificed at day 28, or earlier if they became clinically ill (weight loss more than 20%, ruffled coat or hunching behavior). In the survival study, mice were infected with 500 infectious units of M81 EBV virus and sacrificed when they became clinically ill.

### Anti-CTLA-4, anti-PD-1, and anti-CD3 antibody treatment

OKT3 antibody was purchased from Imgenex (San Diego, CA). Mice received 50 μg of antibody three times per week i.p, starting eleven days after EBV infection and continuing for the remainder of the experiment. The blocking anti-CTLA-4 antibody, ipilimumab, was obtained from the remnants of antibody used to treat cancer patients at the University of Texas MD Anderson Cancer Center. The blocking PD-1 antibody (a-hCD279, clone J116) was obtained from BioXcell. For CTLA-4 and PD-1 blockade, mice were given three times weekly antibody injections (100 μg i.p.), starting five days after cord blood injection (or 10 days after cord blood injection; S2 Fig) and continuing until the end of the experiment. In some experiments (S2 Fig and the survival study), “control” animals were injected with equal amounts of appropriate isotype control antibodies (Human IgG (which is composed of 65% IgG1 subtype), Sigma, and InVivoMAb Mouse IgG1, BioXcell).

### Analysis of EBV infection and tumors

Following euthanasia, grossly visible tumor tissue was carefully excised and weighed. In addition, multiple different organs (including the lungs, transplanted thymus, spleen, pancreas, liver, gall bladder, mesenteric fat and abdominal lymph nodes) were formalin fixed, and then examined using a variety of techniques to determine if animals had persistent EBV infection and/or EBV-positive lymphomas, and to assess the viral protein expression pattern. Analysis performed for all animals included H & E staining, and IHC staining using antibodies directed against CD20 (B cell marker), CD8 (cytotoxic T cell marker), CD4 (helper T cell marker), NFATC1 (activated T cell marker), MECA-79 (a marker for high endothelial venules), and RANTES (a T cell chemokine), and EBV proteins EBNA2, LMP1, BZLF1, and BMRF1. For immunohistochemistry, formalin-fixed, paraffin-embedded tissue sections were deparaffinized and then examined by IHC as previously described [37, 38]. Antibodies used are listed in Table 1. In some animals, EBER *in situ* hybridization studies were performed using the PNA ISH Detection Kit (DakoCytomation).

**Table 1:** Antibodies used for immunohistochemistry.

Antibody	Clone	Manufacturer	Dilution
**CD20**	H1	BD Pharmingen	1:600
**CD4**	4B12	Leica Microsystems	1:80
**LMP1**	CS.1-4	DakoCytomation	1:600
**EBNA2**	PE2	Leica Microsystems	1:100
**BZLF1**	BZ1	Santa Cruz Biotechnology, Inc	1:200
**MECA-79**	Meca-79	Santa Cruz Biotechnology, Inc	1:100
**RANTES**	Ab9679	Abcam	1:200
**BMRF1**	G3-E31	Vector Laboratories	1:200
**CD8**	SP16	Biocare Medical, LLC	1:50
**PD-L1**	E1L3N	Cell Signaling	1:200
**NFATc1**	H-10	Santa Cruz Biotechnology, Inc	1:100

### Flow cytometric analysis

Spleen or tumor tissues were mechanically disrupted and then filtered through a 70μM cell strainer (Fisher Scientific) to generate a single cell suspension. The cells were pelleted, washed in PBS, and then diluted into PBS at a concentration 2–4 million/ml. Staining was performed using fluorescently-labeled monoclonal antibodies specific for human cell surface markers, and compared to isotype-matched negative control antibodies used for fluorescence minus one (FMO) conditions. Antibodies used for staining were purchased from BioLegend, and included the following: anti-HLA-A,B,C (clone W6/32), anti-CD45 (clone HI30), anti-CD3 (clone OKT3), anti-CD19 (clone HIB19), anti-CD20 (clone 2H7), anti-PD-L1 (clone 29E.2A3), anti-PD-L2 (clone 24F.10C12), anti-PD-1 (clone EH12.2H7), and anti-CTLA-4 (clone BNI3 from BD Biosciences). Compensation was performed using single color controls, and staining was detected using an LSRII flow cytometer (BD Biosciences). Flow cytometric data analysis was performed using FlowJo software.

### IFN-γ ELISA

Mice were injected with uninfected or M81-infected cord blood cells, and treated with anti-CTLA-4 and anti-PD-1, or isotype control Abs. Four weeks post injection, T cells were purified from splenocytes via magnetic bead-mediated depletion of non T-cell populations with the Pan-T cell Isolation Kit (Miltenyi Biotec; Auburn, CA) following the manufacturer’s protocol. The purified T cells were incubated in culture medium containing recombinant human IL-2 for 72 hours, then exposed to autologous cord blood mononuclear cells that were pulsed with vehicle control or the indicated concentrations of EBV-derived peptides (PepTivator EBV Consensus, Miltenyi Biotec; Auburn, CA); this peptide pool is derived from 13 different lytic and latent EBV proteins including LMP2a, BRLF1, BMLF1, LMP1, BERF3, BERF1, BERF2, BALF2, BMRF1, BZLF1, BNRF1, EBNA1 and gp350). Alternatively the cord mononuclear cells were pulsed with a peptide pool derived from two different CMV proteins including pp65 and IE1 (Miltenyi Biotec; Auburn, CA), or treated with no peptides. The T cell to APC ratio was 1:1. Supernatants were collected after 24h and analyzed for IFN-γ via sandwich ELISA (Biolegend; San Diego, CA).

### Study approval

All animal work experiments were approved by the University of Wisconsin-Madison Institutional Animal Care and Use Committee (IACUC) and conducted in accordance with the NIH Guide for the care and use of laboratory according to the manufacturer’s protocol as previously described [38].

### Statistics

The p value was calculated using the Wilcoxon rank sum test using Mstat Software (http://mcardle.wisc.edu/mstat/index.html) except in Fig 5A, where the p value was calculated by a 2-tailed student's t test.

## Supporting Information

S1 Fig M81 strain EBV infection induces PD-L1 and PD-L2 expression on lymphoma cells in cord blood-humanized mice. B cells isolated from lymphoma and spleen of an M81-strain EBV-infected cord blood humanized-mouse were stained with antibodies specific for human CD45, CD19, CD20, CD3, PD-L1, PD-L2 or isotype matched negative controls and analyzed by flow cytometry. Samples were gated on lymphocytic cells expressing human CD45, CD19, and CD20 (B cells). Filled histograms show staining for PD-L1 or PD-L2, in comparison to staining of the same population of cells by the isotype control (dashed histograms).
(TIF)

S2 Fig PD-1/CTLA-4 blockade inhibits the growth of EBV-induced lymphomas in cord blood-humanized mice when started 10 days after injection of EBV-infected cells. Mice were treated with anti-PD-1/CTLA-4 ab or isotype control ab as indicated starting 10 days post-injection of EBV-infected cord blood cells. Two different experiments were performed (using two different sets of cord blood), with a total of 11 mice per condition. Mice were euthanized 4 weeks after cord blood injection and grossly visible tumors were weighed. The tumor weight is shown for each condition (normalized to the average tumor weight of isotype control treated animals).
(TIF)

S3 Fig T cells isolated from uninfected cord blood-humanized mice do not respond to EBV or CMV peptides. Human T cells were harvested at 4 weeks post-injection from spleens of uninfected cord-blood humanized mice (using the same donor shown in Fig 5A). The T cells were incubated for 72 hr in medium containing IL-2, then exposed to autologous umbilical cord mononuclear cells in the presence of vehicle control, a mixture of synthetic EBV peptides (“EBV peptide”), or a mixture of CMV peptides (“CMV peptide”). In parallel, the T cells were incubated with an anti-CD3 antibody (OKT3) as a positive control to ensure that they were able to respond. After 24 hr, IFN-γ secreted into the culture supernatant was quantified by ELISA. The results show the means of 3 replicates for each condition with error bars indicating the standard deviations.
(TIF)

## References

[1] Vereide D, Sugden B. Insights into the evolution of lymphomas induced by Epstein-Barr virus. Advances in cancer research. 2010;108:1–19. 10.1016/B978-0-12-380888-2.00001-7 21034964

[2] Vereide DT, Sugden B. Lymphomas differ in their dependence on Epstein-Barr virus. Blood. 2011;117(6):1977–85. 10.1182/blood-2010-05-285791 21088132 PMC3056644

[3] Hatton OL, Harris-Arnold A, Schaffert S, Krams SM, Martinez OM. The interplay between Epstein-Barr virus and B lymphocytes: implications for infection, immunity, and disease. Immunol Res. 2014;58(2–3):268–76. 10.1007/s12026-014-8496-1 24619311 PMC4199828

[4] Houldcroft CJ, Kellam P. Host genetics of Epstein-Barr virus infection, latency and disease. Rev Med Virol. 2015;25(2):71–84. 10.1002/rmv.1816 25430668 PMC4407908

[5] Manzo T, Heslop HE, Rooney CM. Antigen-specific T cell therapies for cancer. Hum Mol Genet. 2015. 10.1093/hmg/ddv270 PMC4572005 26160910

[6] Adhikary D, Behrends U, Boerschmann H, Pfunder A, Burdach S, Moosmann A, et al Immunodominance of lytic cycle antigens in Epstein-Barr virus-specific CD4+ T cell preparations for therapy. PLoS One. 2007;2(7):e583 17611619 10.1371/journal.pone.0000583 PMC1894652

[7] Merlo A, Turrini R, Dolcetti R, Martorelli D, Muraro E, Comoli P, et al The interplay between Epstein-Barr virus and the immune system: a rationale for adoptive cell therapy of EBV-related disorders. Haematologica. 2010;95(10):1769–77. 10.3324/haematol.2010.023689 20421267 PMC2948104

[8] Agathanggelou A, Niedobitek G, Chen R, Nicholls J, Yin W, Young LS. Expression of immune regulatory molecules in Epstein-Barr virus-associated nasopharyngeal carcinomas with prominent lymphoid stroma. Evidence for a functional interaction between epithelial tumor cells and infiltrating lymphoid cells. Am J Pathol. 1995;147(4):1152–60. 7573360 PMC1871000

[9] Huang YT, Sheen TS, Chen CL, Lu J, Chang Y, Chen JY, et al Profile of cytokine expression in nasopharyngeal carcinomas: a distinct expression of interleukin 1 in tumor and CD4+ T cells. Cancer Res. 1999;59(7):1599–605. 10197635

[10] Lau KM, Cheng SH, Lo KW, Lee SA, Woo JK, van Hasselt CA, et al Increase in circulating Foxp3+CD4+CD25(high) regulatory T cells in nasopharyngeal carcinoma patients. Br J Cancer. 2007;96(4):617–22. 17262084 10.1038/sj.bjc.6603580 PMC2360054

[11] Marshall NA, Vickers MA, Barker RN. Regulatory T cells secreting IL-10 dominate the immune response to EBV latent membrane protein 1. J Immunol. 2003;170(12):6183–9. 12794149 10.4049/jimmunol.170.12.6183

[12] Alvaro T, Lejeune M, Salvado MT, Bosch R, Garcia JF, Jaen J, et al Outcome in Hodgkin's lymphoma can be predicted from the presence of accompanying cytotoxic and regulatory T cells. Clin Cancer Res. 2005;11(4):1467–73. 15746048 10.1158/1078-0432.CCR-04-1869

[13] Chen L, Han X. Anti-PD-1/PD-L1 therapy of human cancer: past, present, and future. J Clin Invest. 2015;125(9):3384–91. 10.1172/JCI80011 26325035 PMC4588282

[14] Alegre ML, Frauwirth KA, Thompson CB. T-cell regulation by CD28 and CTLA-4. Nat Rev Immunol. 2001;1(3):220–8. 11905831 10.1038/35105024

[15] McDermott DF, Atkins MB. PD-1 as a potential target in cancer therapy. Cancer Med. 2013;2(5):662–73. 10.1002/cam4.106 24403232 PMC3892798

[16] Boussiotis VA, Chatterjee P, Li L. Biochemical signaling of PD-1 on T cells and its functional implications. Cancer J. 2014;20(4):265–71. 10.1097/PPO.0000000000000059 25098287 PMC4151049

[17] Pardoll DM. The blockade of immune checkpoints in cancer immunotherapy. Nat Rev Cancer. 2012;12(4):252–64. 10.1038/nrc3239 22437870 PMC4856023

[18] Durand-Panteix S, Farhat M, Youlyouz-Marfak I, Rouaud P, Ouk-Martin C, David A, et al B7-H1, which represses EBV-immortalized B cell killing by autologous T and NK cells, is oppositely regulated by c-Myc and EBV latency III program at both mRNA and secretory lysosome levels. J Immunol. 2012;189(1):181–90. 10.4049/jimmunol.1102277 22661084

[19] Rodriguez-Garcia M, Porichis F, de Jong OG, Levi K, Diefenbach TJ, Lifson JD, et al Expression of PD-L1 and PD-L2 on human macrophages is up-regulated by HIV-1 and differentially modulated by IL-10. J Leukoc Biol. 2011;89(4):507–15. 10.1189/jlb.0610327 21097698 PMC3058820

[20] Fang W, Zhang J, Hong S, Zhan J, Chen N, Qin T, et al EBV-driven LMP1 and IFN-gamma up-regulate PD-L1 in nasopharyngeal carcinoma: Implications for oncotargeted therapy. Oncotarget. 2014;5(23):12189–202. 25361008 10.18632/oncotarget.2608 PMC4322961

[21] Green MR, Rodig S, Juszczynski P, Ouyang J, Sinha P, O'Donnell E, et al Constitutive AP-1 activity and EBV infection induce PD-L1 in Hodgkin lymphomas and posttransplant lymphoproliferative disorders: implications for targeted therapy. Clin Cancer Res. 2012;18(6):1611–8. 10.1158/1078-0432.CCR-11-1942 22271878 PMC3321508

[22] Romano A, Conticello C, Cavalli M, Vetro C, La Fauci A, Parrinello NL, et al Immunological dysregulation in multiple myeloma microenvironment. Biomed Res Int. 2014;2014:198539 10.1155/2014/198539 25013764 PMC4071780

[23] Quan L, Chen X, Liu A, Zhang Y, Guo X, Yan S, et al PD-1 Blockade Can Restore Functions of T-Cells in Epstein-Barr Virus-Positive Diffuse Large B-Cell Lymphoma In Vitro. PLoS One. 2015;10(9):e0136476 10.1371/journal.pone.0136476 26361042 PMC4567291

[24] Larsen M, Sauce D, Deback C, Arnaud L, Mathian A, Miyara M, et al Exhausted cytotoxic control of Epstein-Barr virus in human lupus. PLoS Pathog. 2011;7(10):e1002328 10.1371/journal.ppat.1002328 22028659 PMC3197610

[25] Hodi FS, O'Day SJ, McDermott DF, Weber RW, Sosman JA, Haanen JB, et al Improved survival with ipilimumab in patients with metastatic melanoma. N Engl J Med. 2010;363(8):711–23. 10.1056/NEJMoa1003466 20525992 PMC3549297

[26] Sharma P, Wagner K, Wolchok JD, Allison JP. Novel cancer immunotherapy agents with survival benefit: recent successes and next steps. Nat Rev Cancer. 2011;11(11):805–12. 10.1038/nrc3153 22020206 PMC3426440

[27] Ansell SM, Lesokhin AM, Borrello I, Halwani A, Scott EC, Gutierrez M, et al PD-1 blockade with nivolumab in relapsed or refractory Hodgkin's lymphoma. N Engl J Med. 2015;372(4):311–9. 10.1056/NEJMoa1411087 25482239 PMC4348009

[28] Kline J, Bishop MR. Update on checkpoint blockade therapy for lymphoma. J Immunother Cancer. 2015;3:33 10.1186/s40425-015-0079-8 26199729 PMC4509696

[29] Topalian SL, Hodi FS, Brahmer JR, Gettinger SN, Smith DC, McDermott DF, et al Safety, activity, and immune correlates of anti-PD-1 antibody in cancer. N Engl J Med. 2012;366(26):2443–54. 10.1056/NEJMoa1200690 22658127 PMC3544539

[30] Hamid O, Robert C, Daud A, Hodi FS, Hwu WJ, Kefford R, et al Safety and tumor responses with lambrolizumab (anti-PD-1) in melanoma. N Engl J Med. 2013;369(2):134–44. 10.1056/NEJMoa1305133 23724846 PMC4126516

[31] Topalian SL, Sznol M, McDermott DF, Kluger HM, Carvajal RD, Sharfman WH, et al Survival, durable tumor remission, and long-term safety in patients with advanced melanoma receiving nivolumab. J Clin Oncol. 2014;32(10):1020–30. 10.1200/JCO.2013.53.0105 24590637 PMC4811023

[32] Larkin J, Chiarion-Sileni V, Gonzalez R, Grob JJ, Cowey CL, Lao CD, et al Combined Nivolumab and Ipilimumab or Monotherapy in Untreated Melanoma. N Engl J Med. 2015;373(1):23–34. 10.1056/NEJMoa1504030 26027431 PMC5698905

[33] Gajewski TF, Schreiber H, Fu YX. Innate and adaptive immune cells in the tumor microenvironment. Nat Immunol. 2013;14(10):1014–22. 10.1038/ni.2703 24048123 PMC4118725

[34] Boussiotis VA. Somatic mutations and immunotherapy outcome with CTLA-4 blockade in melanoma. N Engl J Med. 2014;371(23):2230–2. 10.1056/NEJMe1413061 25409261 PMC4456677

[35] Yadav M, Jhunjhunwala S, Phung QT, Lupardus P, Tanguay J, Bumbaca S, et al Predicting immunogenic tumour mutations by combining mass spectrometry and exome sequencing. Nature. 2014;515(7528):572–6. 10.1038/nature14001 25428506

[36] Ma SD, Xu X, Plowshay J, Ranheim EA, Burlingham WJ, Jensen JL, et al LMP1-deficient Epstein-Barr virus mutant requires T cells for lymphomagenesis. J Clin Invest. 2015;125(1):304–15. 10.1172/JCI76357 25485679 PMC4382240

[37] Ma SD, Yu X, Mertz JE, Gumperz JE, Reinheim E, Zhou Y, et al An Epstein-Barr Virus (EBV) mutant with enhanced BZLF1 expression causes lymphomas with abortive lytic EBV infection in a humanized mouse model. J Virol. 2012;86(15):7976–87. 10.1128/JVI.00770-12 22623780 PMC3421695

[38] Ma SD, Hegde S, Young KH, Sullivan R, Rajesh D, Zhou Y, et al A new model of Epstein-Barr virus infection reveals an important role for early lytic viral protein expression in the development of lymphomas. J Virol. 2011;85(1):165–77. 10.1128/JVI.01512-10 20980506 PMC3014199

[39] Strowig T, Gurer C, Ploss A, Liu YF, Arrey F, Sashihara J, et al Priming of protective T cell responses against virus-induced tumors in mice with human immune system components. J Exp Med. 2009;206(6):1423–34. 10.1084/jem.20081720 19487422 PMC2715061

[40] Yajima M, Imadome K, Nakagawa A, Watanabe S, Terashima K, Nakamura H, et al T cell-mediated control of Epstein-Barr virus infection in humanized mice. J Infect Dis. 2009;200(10):1611–5. 10.1086/644644 19832115

[41] Brinkman CC, Peske JD, Engelhard VH. Peripheral tissue homing receptor control of naive, effector, and memory CD8 T cell localization in lymphoid and non-lymphoid tissues. Front Immunol. 2013;4:241 10.3389/fimmu.2013.00241 23966998 PMC3746678

[42] Peske JD, Woods AB, Engelhard VH. Control of CD8 T-Cell Infiltration into Tumors by Vasculature and Microenvironment. Advances in cancer research. 2015;128:263–307. 10.1016/bs.acr.2015.05.001 26216636 PMC4638417

[43] Peske JD, Thompson ED, Gemta L, Baylis RA, Fu YX, Engelhard VH. Effector lymphocyte-induced lymph node-like vasculature enables naive T-cell entry into tumours and enhanced anti-tumour immunity. Nat Commun. 2015;6:7114 10.1038/ncomms8114 25968334 PMC4435831

[44] Pan M, Winslow MM, Chen L, Kuo A, Felsher D, Crabtree GR. Enhanced NFATc1 nuclear occupancy causes T cell activation independent of CD28 costimulation. J Immunol. 2007;178(7):4315–21. 17371988 10.4049/jimmunol.178.7.4315

[45] Uchihara JN, Krensky AM, Matsuda T, Kawakami H, Okudaira T, Masuda M, et al Transactivation of the CCL5/RANTES gene by Epstein-Barr virus latent membrane protein 1. Int J Cancer. 2005;114(5):747–55. 15609310 10.1002/ijc.20784

[46] Katz BZ, Andiman WA, Eastman R, Martin K, Miller G. Infection with two genotypes of Epstein-Barr virus in an infant with AIDS and lymphoma of the central nervous system. J Infect Dis. 1986;153(3):601–4. 3005427 10.1093/infdis/153.3.601

[47] Carbone A, Cesarman E, Spina M, Gloghini A, Schulz TF. HIV-associated lymphomas and gamma-herpesviruses. Blood. 2009;113(6):1213–24. 10.1182/blood-2008-09-180315 18955561

[48] Martis N, Mounier N. Hodgkin lymphoma in patients with HIV infection: a review. Curr Hematol Malig Rep. 2012;7(3):228–34. 10.1007/s11899-012-0125-2 22547166

[49] Gloghini A, Dolcetti R, Carbone A. Lymphomas occurring specifically in HIV-infected patients: from pathogenesis to pathology. Semin Cancer Biol. 2013;23(6):457–67. 10.1016/j.semcancer.2013.08.004 23999127

[50] Papadopoulos EB, Ladanyi M, Emanuel D, Mackinnon S, Boulad F, Carabasi MH, et al Infusions of donor leukocytes to treat Epstein-Barr virus-associated lymphoproliferative disorders after allogeneic bone marrow transplantation. N Engl J Med. 1994;330(17):1185–91. 8093146 10.1056/NEJM199404283301703

[51] Shapiro RS, McClain K, Frizzera G, Gajl-Peczalska KJ, Kersey JH, Blazar BR, et al Epstein-Barr virus associated B cell lymphoproliferative disorders following bone marrow transplantation. Blood. 1988;71(5):1234–43. 2833957

[52] Chattopadhyay PK, Chelimo K, Embury PB, Mulama DH, Sumba PO, Gostick E, et al Holoendemic malaria exposure is associated with altered Epstein-Barr virus-specific CD8(+) T-cell differentiation. J Virol. 2013;87(3):1779–88. 10.1128/JVI.02158-12 23175378 PMC3554182

[53] Piriou E, Asito AS, Sumba PO, Fiore N, Middeldorp JM, Moormann AM, et al Early age at time of primary Epstein-Barr virus infection results in poorly controlled viral infection in infants from Western Kenya: clues to the etiology of endemic Burkitt lymphoma. J Infect Dis. 2012;205(6):906–13. 10.1093/infdis/jir872 22301635 PMC3282570

[54] Asano N, Yamamoto K, Tamaru J, Oyama T, Ishida F, Ohshima K, et al Age-related Epstein-Barr virus (EBV)-associated B-cell lymphoproliferative disorders: comparison with EBV-positive classic Hodgkin lymphoma in elderly patients. Blood. 2009;113(12):2629–36. 10.1182/blood-2008-06-164806 19075188

[55] Deacon EM, Pallesen G, Niedobitek G, Crocker J, Brooks L, Rickinson AB, et al Epstein-Barr virus and Hodgkin's disease: transcriptional analysis of virus latency in the malignant cells. J Exp Med. 1993;177(2):339–49. 8381153 10.1084/jem.177.2.339 PMC2190903

[56] Young LS, Dawson CW, Clark D, Rupani H, Busson P, Tursz T, et al Epstein-Barr virus gene expression in nasopharyngeal carcinoma. J Gen Virol. 1988;69 (Pt 5):1051–65. 2836550 10.1099/0022-1317-69-5-1051

[57] Gottschalk S, Edwards OL, Sili U, Huls MH, Goltsova T, Davis AR, et al Generating CTLs against the subdominant Epstein-Barr virus LMP1 antigen for the adoptive immunotherapy of EBV-associated malignancies. Blood. 2003;101(5):1905–12. 12411306 10.1182/blood-2002-05-1514

[58] Haigh TA, Lin X, Jia H, Hui EP, Chan AT, Rickinson AB, et al EBV latent membrane proteins (LMPs) 1 and 2 as immunotherapeutic targets: LMP-specific CD4+ cytotoxic T cell recognition of EBV-transformed B cell lines. J Immunol. 2008;180(3):1643–54. 18209060 10.4049/jimmunol.180.3.1643

[59] Le Clorennec C, Ouk TS, Youlyouz-Marfak I, Panteix S, Martin CC, Rastelli J, et al Molecular basis of cytotoxicity of Epstein-Barr virus (EBV) latent membrane protein 1 (LMP1) in EBV latency III B cells: LMP1 induces type II ligand-independent autoactivation of CD95/Fas with caspase 8-mediated apoptosis. J Virol. 2008;82(13):6721–33. 10.1128/JVI.02250-07 18448526 PMC2447067

[60] Brooks JM, Lee SP, Leese AM, Thomas WA, Rowe M, Rickinson AB. Cyclical expression of EBV latent membrane protein 1 in EBV-transformed B cells underpins heterogeneity of epitope presentation and CD8+ T cell recognition. J Immunol. 2009;182(4):1919–28. 10.4049/jimmunol.0713607 19201845

[61] Cancer Genome Atlas Research N. Comprehensive molecular characterization of gastric adenocarcinoma. Nature. 2014;513(7517):202–9. 10.1038/nature13480 25079317 PMC4170219

[62] Hamid O, Schmidt H, Nissan A, Ridolfi L, Aamdal S, Hansson J, et al A prospective phase II trial exploring the association between tumor microenvironment biomarkers and clinical activity of ipilimumab in advanced melanoma. J Transl Med. 2011;9:204 10.1186/1479-5876-9-204 22123319 PMC3239318

[63] White RE, Ramer PC, Naresh KN, Meixlsperger S, Pinaud L, Rooney C, et al EBNA3B-deficient EBV promotes B cell lymphomagenesis in humanized mice and is found in human tumors. J Clin Invest. 2012;122(4):1487–502. 10.1172/JCI58092 22406538 PMC3314448

[64] Levitskaya J, Coram M, Levitsky V, Imreh S, Steigerwald-Mullen PM, Klein G, et al Inhibition of antigen processing by the internal repeat region of the Epstein-Barr virus nuclear antigen-1. Nature. 1995;375(6533):685–8. 7540727 10.1038/375685a0

[65] Yin Y, Manoury B, Fahraeus R. Self-inhibition of synthesis and antigen presentation by Epstein-Barr virus-encoded EBNA1. Science. 2003;301(5638):1371–4. 12958359 10.1126/science.1088902

[66] Kyi C, Postow MA. Checkpoint blocking antibodies in cancer immunotherapy. FEBS Lett. 2014;588(2):368–76. 10.1016/j.febslet.2013.10.015 24161671

[67] Tsai MH, Raykova A, Klinke O, Bernhardt K, Gartner K, Leung CS, et al Spontaneous lytic replication and epitheliotropism define an Epstein-Barr virus strain found in carcinomas. Cell Rep. 2013;5(2):458–70. 10.1016/j.celrep.2013.09.012 24120866

[68] Dirmeier U, Neuhierl B, Kilger E, Reisbach G, Sandberg ML, Hammerschmidt W. Latent membrane protein 1 is critical for efficient growth transformation of human B cells by epstein-barr virus. Cancer Res. 2003;63(11):2982–9. 12782607

