# Supplementary figures and images for: PD-1/CTLA-4 Blockade Inhibits Epstein-Barr Virus-Induced Lymphoma Growth in a Cord Blood Humanized-Mouse Model
Source: PLoS Pathog. 2016 May 17;12(5):e1005642. doi: 10.1371/journal.ppat.1005642 (PMC4871349; doi:10.1371/journal.ppat.1005642)

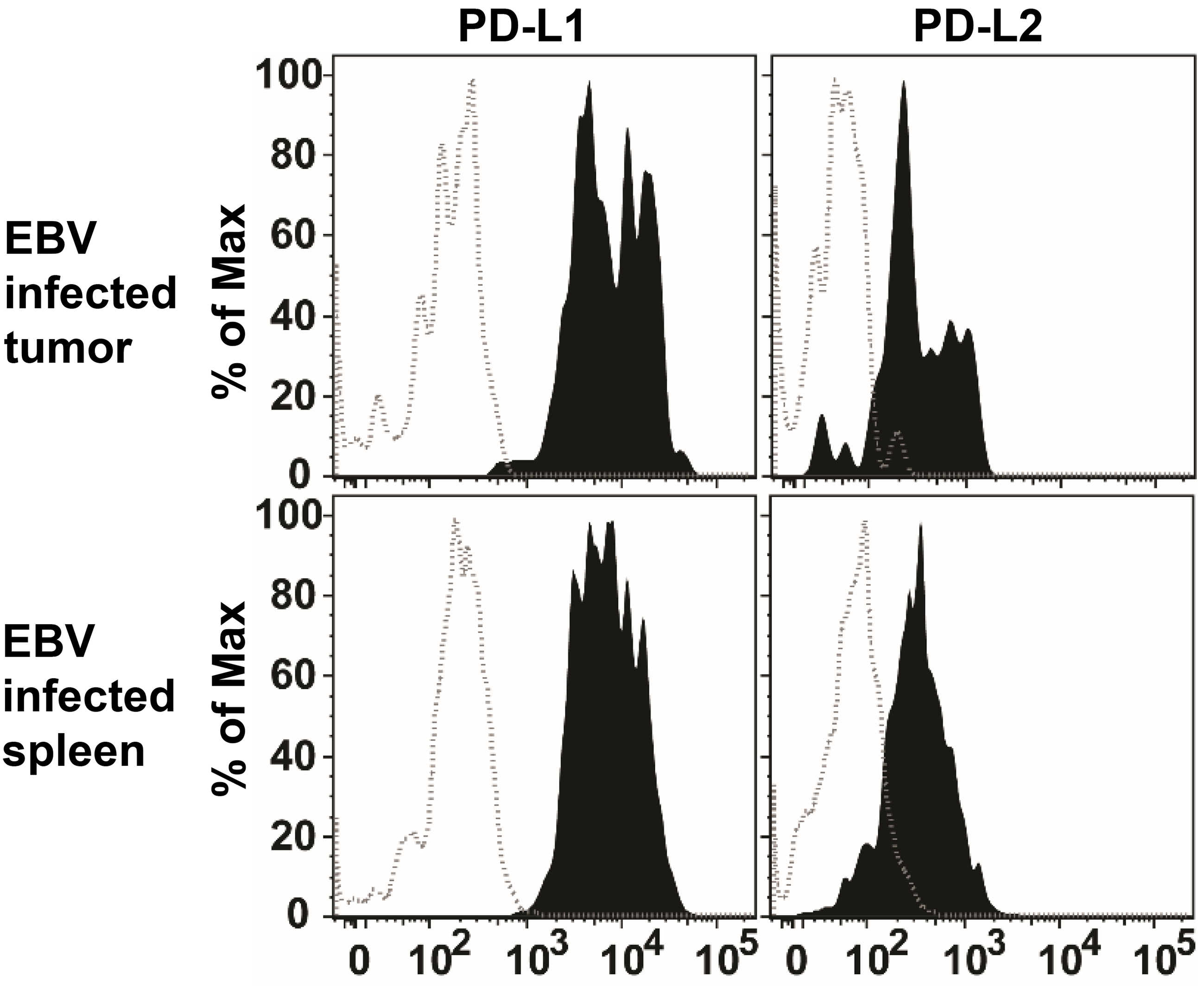

Supplement: S1 Fig — B cells isolated from lymphoma and spleen of an M81-strain EBV-infected cord blood humanized-mouse were stained with antibodies specific for human CD45, CD19, CD20, CD3, PD-L1, PD-L2 or isotype matched negative controls and analyzed by flow cytometry. Samples were gated on lymphocytic cells expressing human CD45, CD19, and CD20 (B cells). Filled histograms show staining for PD-L1 or PD-L2, in comparison to staining of the same population of cells by the isotype control (dashed histograms). (TIF) [file ppat.1005642.s001.tif]

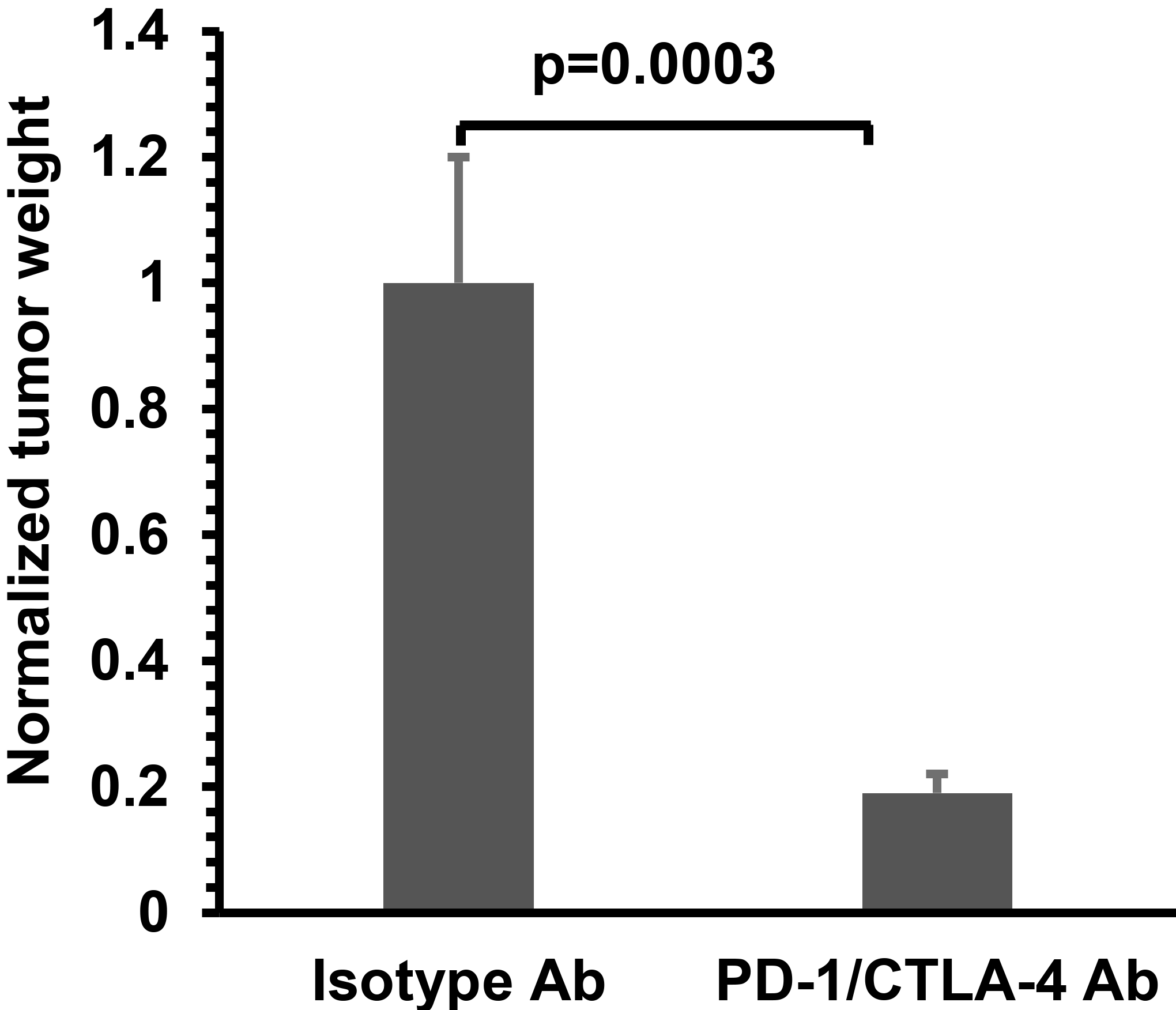

Supplement: S2 Fig — Mice were treated with anti-PD-1/CTLA-4 ab or isotype control ab as indicated starting 10 days post-injection of EBV-infected cord blood cells. Two different experiments were performed (using two different sets of cord blood), with a total of 11 mice per condition. Mice were euthanized 4 weeks after cord blood injection and grossly visible tumors were weighed. The tumor weight is shown for each condition (normalized to the average tumor weight of isotype control treated animals). (TIF) [file ppat.1005642.s002.tif]

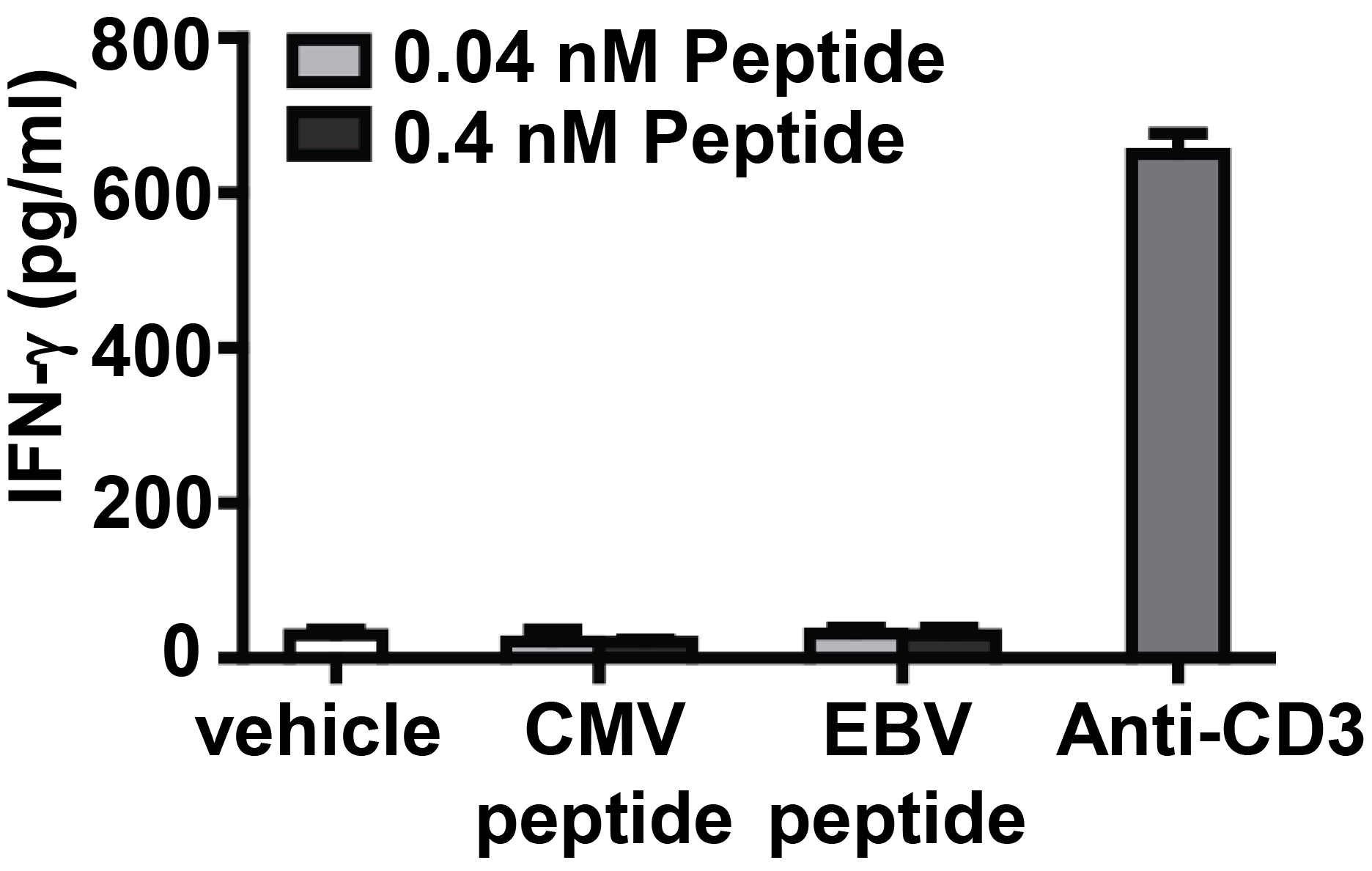

Supplement: S3 Fig — Human T cells were harvested at 4 weeks post-injection from spleens of uninfected cord-blood humanized mice (using the same donor shown in Fig 5A). The T cells were incubated for 72 hr in medium containing IL-2, then exposed to autologous umbilical cord mononuclear cells in the presence of vehicle control, a mixture of synthetic EBV peptides (“EBV peptide”), or a mixture of CMV peptides (“CMV peptide”). In parallel, the T cells were incubated with an anti-CD3 antibody (OKT3) as a positive control to ensure that they were able to respond. After 24 hr, IFN-γ secreted into the culture supernatant was quantified by ELISA. The results show the means of 3 replicates for each condition with error bars indicating the standard deviations. (TIF) [file ppat.1005642.s003.tif]
